# A network medicine framework for multi-modal data integration in therapeutic target discovery

**DOI:** 10.1038/s42004-026-02049-9

**Published:** 2026-05-06

**Authors:** Greta Baltušytė, Isaac J. D. Toleman, James O. Jones, Sarah J. Welsh, Grant D. Stewart, Thomas J. Mitchell, Kourosh Saeb-Parsy, Namshik Han

**Affiliations:** 1 Milner Therapeutics Institute, University of Cambridge, Cambridge, UK; 2 Department of Surgery, University of Cambridge, and Cambridge NIHR Biomedical Research Centre, Cambridge, UK; 3 Cambridge Stem Cell Institute, University of Cambridge, Cambridge, UK; 4 Cambridge Centre for AI in Medicine, University of Cambridge, Cambridge, UK; 5 Department of Oncology, University of Cambridge, Cambridge, UK; 6 Cambridge University Hospitals NHS Foundation Trust, Cambridge, UK; 7 Wellcome Trust Sanger Institute, Wellcome Genome Campus, Cambridge, UK; 8 Department of Quantum Information, Institute for Convergence Research and Education in Advanced Technology and Engineering, Yonsei University, Seoul, Republic of Korea; 9 Department of Nano Biomedical Engineering (NanoBME), Advanced Science Institute, Yonsei University, Seoul, Republic of Korea; 10 Center for Nanomedicine, Institute for Basic Science (IBS), Seoul, Republic of Korea

**Keywords:** Target identification, Cheminformatics, Computational chemistry, Target validation

## Abstract

The high cost and attrition rate of drug development underscore the need for more effective strategies for therapeutic target discovery. Here, we present a network medicine-based machine learning framework that integrates single-cell transcriptomics, bulk multi-omic profiles, genome-wide CRISPR perturbation screens, and protein-protein interaction networks to systematically prioritise disease-specific targets. Applied to clear cell renal cell carcinoma, the framework successfully recovered established targets and predicted five therapeutic candidates, with subsequent in vitro validation demonstrating that among these, *ENO2* inhibition had the strongest anti-tumour effect, followed by *LRRK2*, a repurposing candidate with phase III Parkinson’s disease inhibitors. The proposed approach advances target discovery by moving beyond single-feature, single-modality heuristics to a scalable, machine learning-driven strategy that is generalisable across diseases.

## Abstract (web summary)

The high cost and attrition rate of drug development highlight the urgent need for improved therapeutic target discovery strategies. Here, the authors introduce a network medicine-based machine learning framework that integrates multi-omic data and protein interaction networks, successfully identifying and validating targets for clear cell renal cell carcinoma, including ENO2 and LRRK2.

## Introduction

Targeted therapies have reshaped the clinical management of cancer by enabling selective inhibition of disease-driving molecular alterations, offering improved efficacy and reduced systemic toxicity compared to conventional chemotherapeutics. Yet, despite their transformative potential, the promise of precision oncology remains constrained. A limited pool of actionable targets, tumour heterogeneity and the frequent development of drug resistance continue to restrict the long-term success of targeted interventions, underscoring the need for therapeutic landscape expansion.

Conventional approaches to target discovery have typically relied on single-gene hypotheses, often informed by mutational profiling or differential expression analyses. While these methods have uncovered key oncogenic drivers, they fall short in capturing the interplay of molecular interactions that underpin disease pathophysiology. Mounting evidence suggests that pathologies such as cancer emerge not from isolated molecular events but from the disruption of coordinated gene networks^1–3^. This systems-level perspective has led to the emergence of network medicine, which leverages insights from network theory^4–6^ to model the molecular architecture of disease. Among the most important observations in this field is that disease-associated genes often cluster in the same network neighbourhood, forming a disease module. Empirical studies have confirmed that drug targets tend to reside within or near these modules^7,8^, suggesting that network topology can be harnessed to prioritise therapeutically relevant genes. Accordingly, a wide range of network-based methods have been proposed to identify candidate therapeutic targets and reposition existing drugs, often leveraging metrics such as shortest path, nearest neighbour, and node centrality. For instance, centrality metrics have been used to highlight key hubs in disease-specific protein–protein interaction (PPI) networks^9,10^, while network proximity analyses have demonstrated strong predictive power in drug repurposing and target prioritisation tasks^7,8,11,12^.

**Fig. 1:**
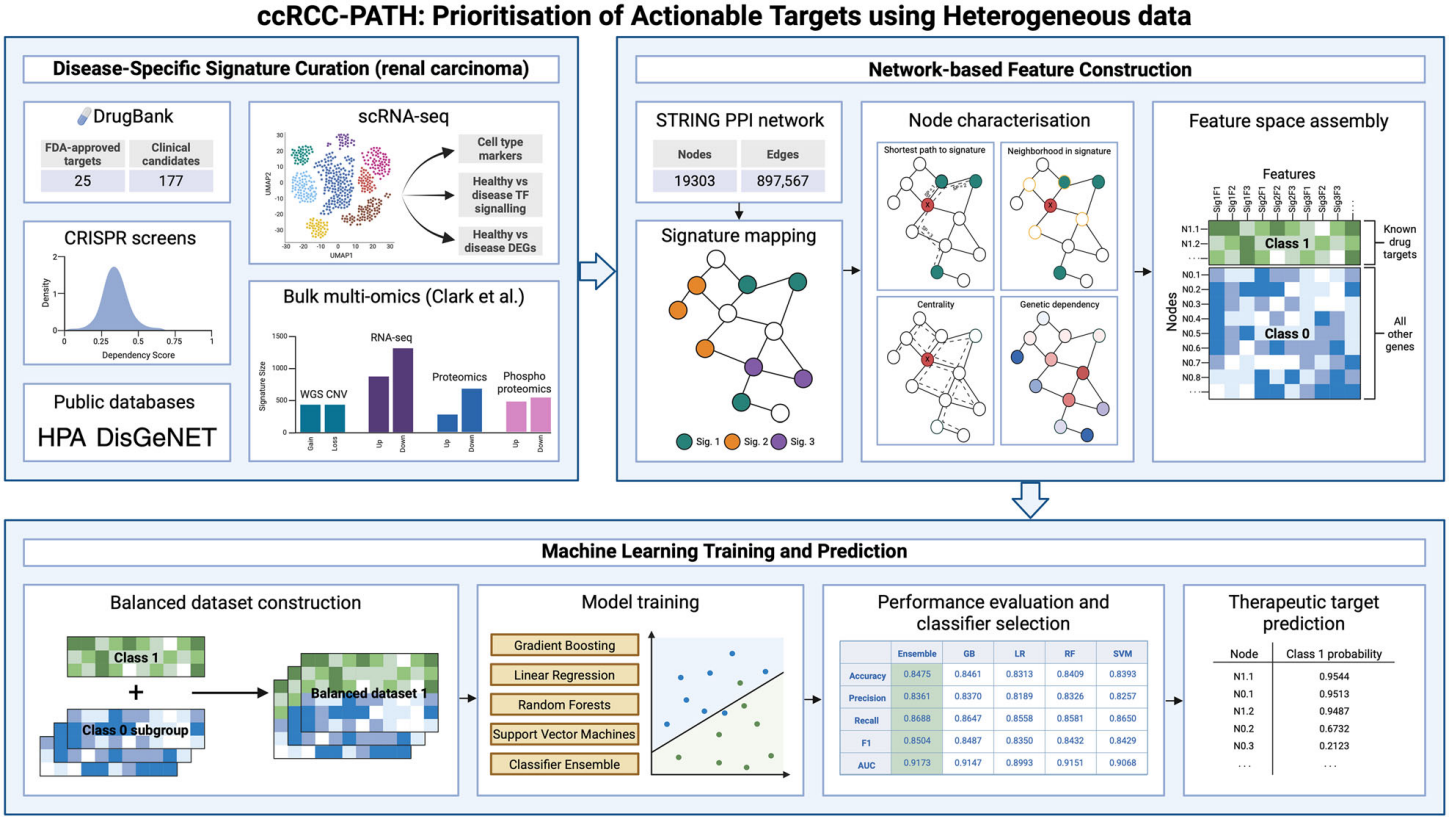
Overview of ccRCC-PATH. Disease-specific gene signatures assembled from DrugBank, single-cell RNA-seq, bulk multi-omics, genome-wide CRISPR perturbation screens, and additional publicly available resources are mapped onto a protein–protein interaction network to derive graph-based descriptors for each gene. The resulting feature space is used to train machine learning models to distinguish known drug targets from all other genes, enabling systematic prioritisation of context-specific therapeutic candidates.

Despite promising advances, most existing methods remain drug-centric, inherently limited by the scope of known compound-target interactions. Moreover, many frameworks operate on a single data modality, overlooking the available wealth of multi-omic and functional genomic data. Here, we introduce PATH (Prioritisation of Actionable Targets using Heterogeneous data), a multimodal machine learning framework that integrates single-cell RNA sequencing, bulk-omics, genome-wide CRISPR knockout data, and PPI networks to systematically profile and prioritise potential therapeutic targets in a disease-specific manner (Fig. 1). Starting from publicly available datasets, we define disease-associated gene signatures, map them onto the human interactome, and extract network-informed descriptors for each gene, including average and minimum shortest-path distances to every signature, PPI neighbourhood composition together with its genetic dependency, and network node centralities. These features are then used to train a class-balanced machine learning framework to distinguish known drug targets from non-targets. Following model evaluation and selection, the final classifier assigns a probability score to each gene, reflecting its predicted relevance to therapeutic intervention in the specific disease context. These scores are then used to generate a genome-wide ranking of candidate targets for downstream prioritisation, experimental validation, and thus novel therapeutic target identification.

We applied this framework to clear-cell renal cell carcinoma (ccRCC; ccRCC-PATH), the predominant subtype of kidney cancer, which accounts for approximately 80% of cases in a cancer that ranks sixth in prevalence in the UK^13^. It arises from proximal tubular epithelial cells (PTECs), with inactivation of the *VHL* tumour suppressor gene on chromosome 3p25 representing a hallmark initiating event^14–16^. In healthy PTECs, *VHL* encodes a component of an E3 ubiquitin ligase complex that targets hypoxia-inducible factors (*HIF-1α* and *HIF-2α*) for degradation. Loss of *VHL* leads to *HIF* accumulation under normoxic conditions, driving metabolic reprogramming, angiogenesis, and immune infiltration – features that collectively underpin the histopathological and molecular phenotype of ccRCC. Although tyrosine kinase inhibitors targeting the VEGF pathway and immune checkpoint inhibitors offer clinical benefit, a substantial proportion of patients exhibit intrinsic or acquired resistance, underscoring the need for alternative therapeutic strategies^17^. Notably, few existing RCC treatments act directly on tumour-intrinsic pathways, with the exception of *HIF* pathway inhibitors such as belzutifan, suggesting additional cell-intrinsic vulnerabilities remain unexploited. Using our framework, we identified and experimentally validated five candidates – *ENO2*, *LRRK2*, *SCARB1*, *HMOX1*, and *TGM2* – whose inhibition significantly impaired tumour cell viability, highlighting their potential as therapeutic targets.

## Results

### Curation of ccRCC-specific gene signatures

To characterise ccRCC at a systems level, we first curated candidate disease-associated gene (CDAG) signatures spanning multiple molecular layers (Fig. 1, Table 1). These comprised gene sets derived from bulk tumour-normal comparisons (RNA-seq, proteomics, phosphoproteomics, and whole-genome sequencing copy number data) as well as ccRCC-related signatures from DisGeNET^18^, the Human Protein Atlas^19^, and DepMap^20^ databases. In parallel, we assembled a reference set of 202 drug target genes (DTGs), consisting of 25 genes targeted by FDA-approved ccRCC therapies and 177 genes under investigation in phase I–IV clinical trials (Supplementary Data 1). As the cell-type-resolved component of this curation, we next analysed single-cell RNA sequencing (scRNA-seq) data from 12 patients in the Li et al. cohort^21^. In that study, multiple anatomical regions were sampled from each donor for transcriptomic profiling, including the tumour core, tumour-normal interface, adjacent normal kidney, peripheral blood, perinephric fat, normal adrenal gland, adrenal metastasis, and tumour thrombus, where available. In the original study, transcriptomic data from all 12 donors were included in the downstream analyses. In contrast, our analysis focused exclusively on the 10 donors with histopathologically confirmed ccRCC, excluding the two non-RCC samples prior to re-clustering. Following quality control, we retained the transcriptomes of 250,331 cells, which were clustered to resolve 16 distinct broad cell types based on canonical marker gene expression and subsequently assessed for their distribution across tissue sites (Supplementary Fig. 1). Tumour cells were annotated based on *CA9* over-expression, a marker absent in healthy renal tissue but strongly induced in ccRCC as a result of *HIF-1α* accumulation^22^.

**Table 1:** Drug target and candidate disease-associated gene signature descriptions

Signature type	Signature data source	Description	Number of signatures
DTGs	DrugBank^78^	Genes targeted by FDA-approved therapeutics for ccRCC, or by drugs that are currently in clinical trials for ccRCC (Supplementary Data 1).	1
CDAGs	Bulk ccRCC data from Clark et al^79^.	Up- and down-regulated transcripts, proteins and phosphoproteins in tumour vs healthy tissue comparisons, as identified through bulk RNA-seq, proteomics, and phosphoproteomics differential abundance analysis; Top 1000 most commonly lost and gained genes identified through WGS data analysis.	8
	scRNA-seq TF activity inference results	Activated and inactivated TFs in tumour vs PT PRAP1+ comparison, determined by differential activity analysis using SCENIC^23^ output.	2
	scRNA-seq Tumour vs PT PRAP1+ DEGs	Up- and down-regulated genes in scRNA-seq tumour vs PT PRAP1+ differential gene expression analysis.	2
	scRNA-seq broad cell-type markers	Up-regulated genes in scRNA-seq differential expression analysis comparing tumour cells, B cells, plasma cells, CD4+ T cells, Tregs, CD8+ T cells, macrophages, monocytes, mast cells, cDC1, cDC2, pDC, NK, endothelium, fibroblasts, PT PRAP1+ epithelium, and the remaining epithelium.	17
	DisGeNET^18^	ccRCC-associated genes (CAGs).	1
	Human Protein Atlas^19^	Favourable and unfavourable ccRCC prognosis genes (FPGs and UPGs).	2
	DepMap^20^	Genes classified as common essentials by DepMap CRISPR or RNAi projects.	1
	'Others'	Background reference signature consisting of genes that do not belong to any of the gene sets outlined above (complement of the curated signature panel).	1
Total			35

To achieve a more refined characterisation of the identified broad cell types, we performed sub-clustering analyses within each major population. A key limitation of current scRNA-seq technologies is the high gene dropout rate, where transcripts from lowly expressed genes are often missed, leading to zero or near-zero read counts. We hypothesised that clustering cells based on inferred transcription factor (TF) activity, rather than directly using UMI data, could help mitigate this issue and provide a more informative broad cell-type sub-clustering output. First, TFs are major determinants of a cell’s phenotype, and so their activity level can provide significant insights into the cell type of a particular cluster. Second, tools such as SCENIC^23^ work as biological dimensionality reduction methods by aggregating the expression levels of a TF and its predicted targets into a single score. Consequently, regulon (defined as a TF and all its predicted targets) activity inference and its subsequent use in scRNA-seq clustering enable the identification of cell clusters based on groups of genes with functional relationships. Therefore, even if some genes in a regulon are not captured during sequencing due to dropout, the presence of other genes in the same regulon allows for more accurate cell identity assignment.

**Fig. 2:**
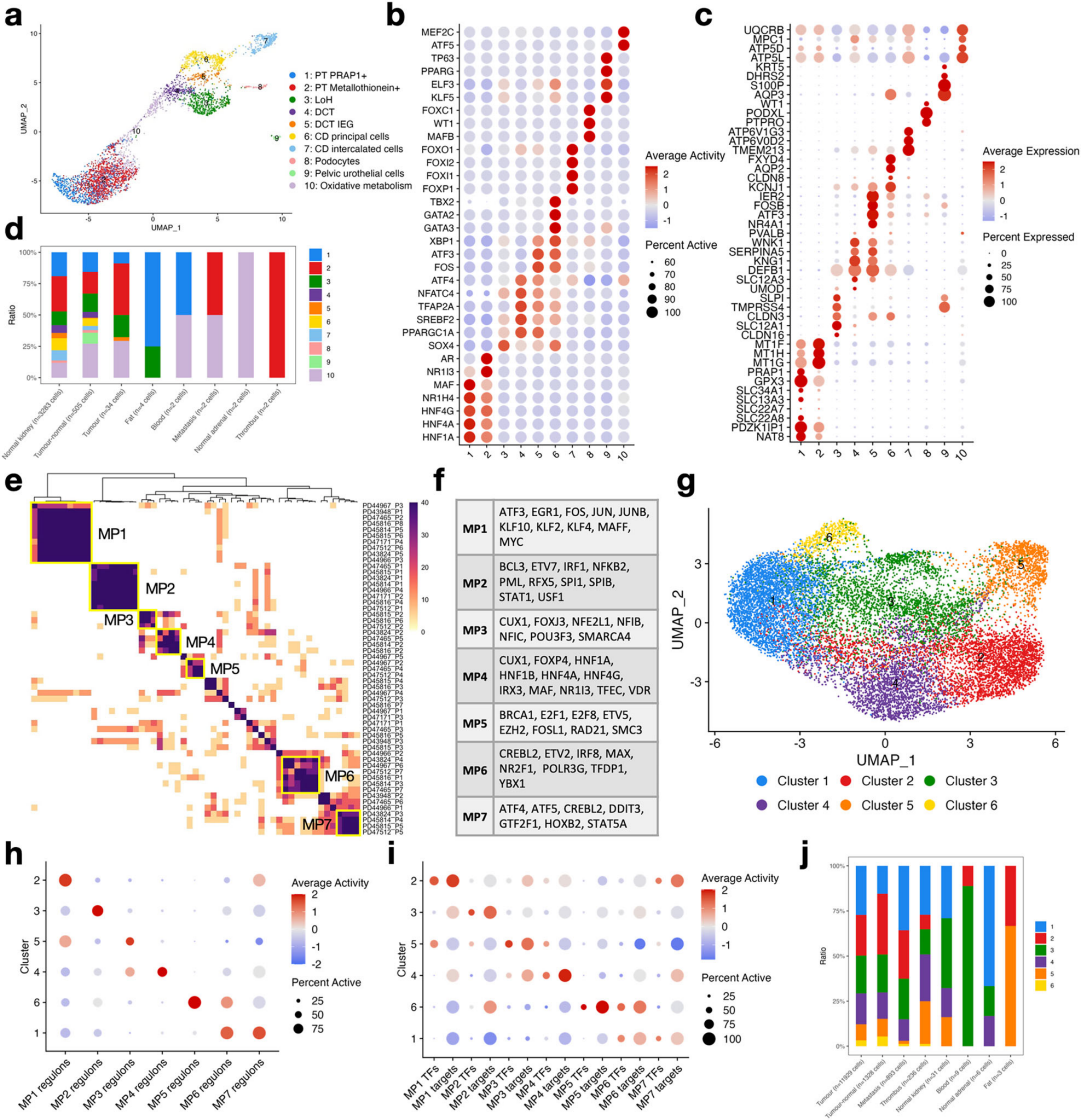
Transcription factor activity-based sub-clustering refines epithelial and tumour cell populations. **a** UMAP visualisation of epithelial cells following sub-clustering based on inferred transcription factor (TF) activities. Per-cell TF activity scores were computed using the SCENIC pipeline, and the resulting regulon activity matrix was used for clustering. Cluster identities were subsequently mapped back onto the scRNA-seq data to assess marker gene expression and validate annotations. **b** Inferred regulon (TF activity) scores across epithelial sub-clusters. **c** Expression of canonical epithelial marker genes across the identified sub-clusters. **d** Distribution of epithelial sub-clusters across anatomical regions sampled for sequencing. **e** Heatmap of 55 tumour transcriptional programmes identified using non-negative matrix factorisation (NMF). Rows and columns represent TF activity programmes across individual patients, with colouring intensity reflecting similarity between programmes. Yellow squares indicate seven transcriptional meta-programmes (MPs) shared across multiple patients. **f** Defining TFs for each of the seven identified tumour meta-programmes. **g** UMAP representation of tumour cells following TF activity-based sub-clustering. **h** Average meta-programme regulon activity across the identified tumour sub-clusters. **i** Average expression of representative TFs and their target genes defining each meta-programme. **j** Distribution of tumour sub-clusters across anatomical regions sampled for sequencing.

TF activity-based sub-clustering was applied systematically to all annotated cell types (Fig. 2, Supplementary Fig. 3). Within the epithelial compartment, this approach revealed 10 cell groups (Fig. 2a–d). These included two proximal tubule (PT) populations, consistent with the predicted *HNF4A*^24^, *HNF4G*^25^, and *MAF*^26^ TF activities (Fig. 2b). Cluster 1 was distinguished by the highest expression of markers characterising all three PT segments^27^ and is hypothesised to represent the cell of origin of ccRCC, while cluster 2 exhibited heightened expression of metallothionein family genes (Fig. 2c). Meanwhile, cluster 3 represents the loop of Henley (LoH), based on elevated levels of genes such as *CLDN3*, *CLDN16*^28^, and *SLC12A1*^29^. Clusters 4 & 5 expressed markers of the distal convoluted tubule (DCT), examples including *WNK1*, *SLC12A3*, and *TFAP2A*^30^ regulon activity (Fig. 2b, c), with cluster 5 also overexpressing immediate-early genes. Furthermore, two clusters were identified as collecting duct (CD) cells, with one corresponding to principal cells (*AQP2*, *GATA2* & *GATA3*^31^) and another to intercalated cells (*ATP6V0D2*, *FOXI1*^32^). Cluster 8 was annotated as a podocyte population given the elevated *PODXL* gene expression (Fig. 2c) and up-regulated *WT1* regulon activity^33^ (Fig. 2b). The pelvic urothelial cluster was annotated based on *AQP3* and *S100P* expression, consistent with the increased activity of *TP63* TF^34^, among others. Lastly, a cluster exhibiting elevated levels of genes involved in oxidative phosphorylation was observed, agreeing with the up-regulated *ATF5*^35^ regulon activity.

In contrast, tumour cell analysis presents a greater challenge due to the uncertainty surrounding the number and characteristics of clusters that one should expect to see. To address this, we applied non-negative matrix factorisation (NMF) to the TF activity profiles of tumour cells for each patient individually^21,36^. This approach facilitated the identification of transcriptional programmes that are shared by multiple patients, thus representing transcriptional ‘meta-programmes’ (MPs). Overall, the outlined analysis of tumour cells yielded a total of 55 transcriptional programmes and 7 transcriptional meta-programmes (Fig. 2e, f), which were then assessed for Reactome^37^ pathway enrichment (Supplementary Fig. 2). MP1 showed enrichment in mRNA translation pathways, while MP2 was distinguished by a significant presence of immune interaction pathways. In contrast, MP3 was linked to a single pathway, namely FOXO-mediated transcription. MP4 displayed enrichment in pathways typically found in tubular kidney cells, including SLC-mediated transmembrane transport and lipid metabolism. MP5 encompassed cell division pathways, while MP6 was associated with mRNA translation, cell division, and several immune pathways. Finally, MP7 was characterised by pathways that are typically up-regulated during starvation. Once meta-programmes were established, tumour cells were clustered. As expected, different clusters are associated with different meta-programmes, with a given cluster expressing at most 2 MPs (Fig. 2g–j).

The outlined TF-based sub-clustering was subsequently applied to the remaining broad cell types (Supplementary Fig. 3, Supplementary Data 2 and 3). Overall, the approach proved to be advantageous, enhancing cluster resolution by partially overcoming the limitations imposed by gene dropout in scRNA-seq data and providing additional insights in the form of inferred TF activities. For instance, within the CD4⁺ T cell compartment, it enabled a clear distinction of Th1, Tfh, and Th17 helper cell subsets – populations that were not resolved in the original study due to the near-complete dropout of both key transcription factors and conventional surface markers typically used to define these subsets. Taken together, in-depth reanalysis of scRNA-seq data yielded 21 gene signatures, which, combined with bulk multi-omic datasets and curated public resources, brought the total number of CDAG signatures to 34 (Table 1). Alongside the DTG reference set, this comprehensive signature collection formed the basis for downstream network-level characterisation of ccRCC.

### Network analysis for characterisation of candidate disease-associated genes

**Fig. 3:**
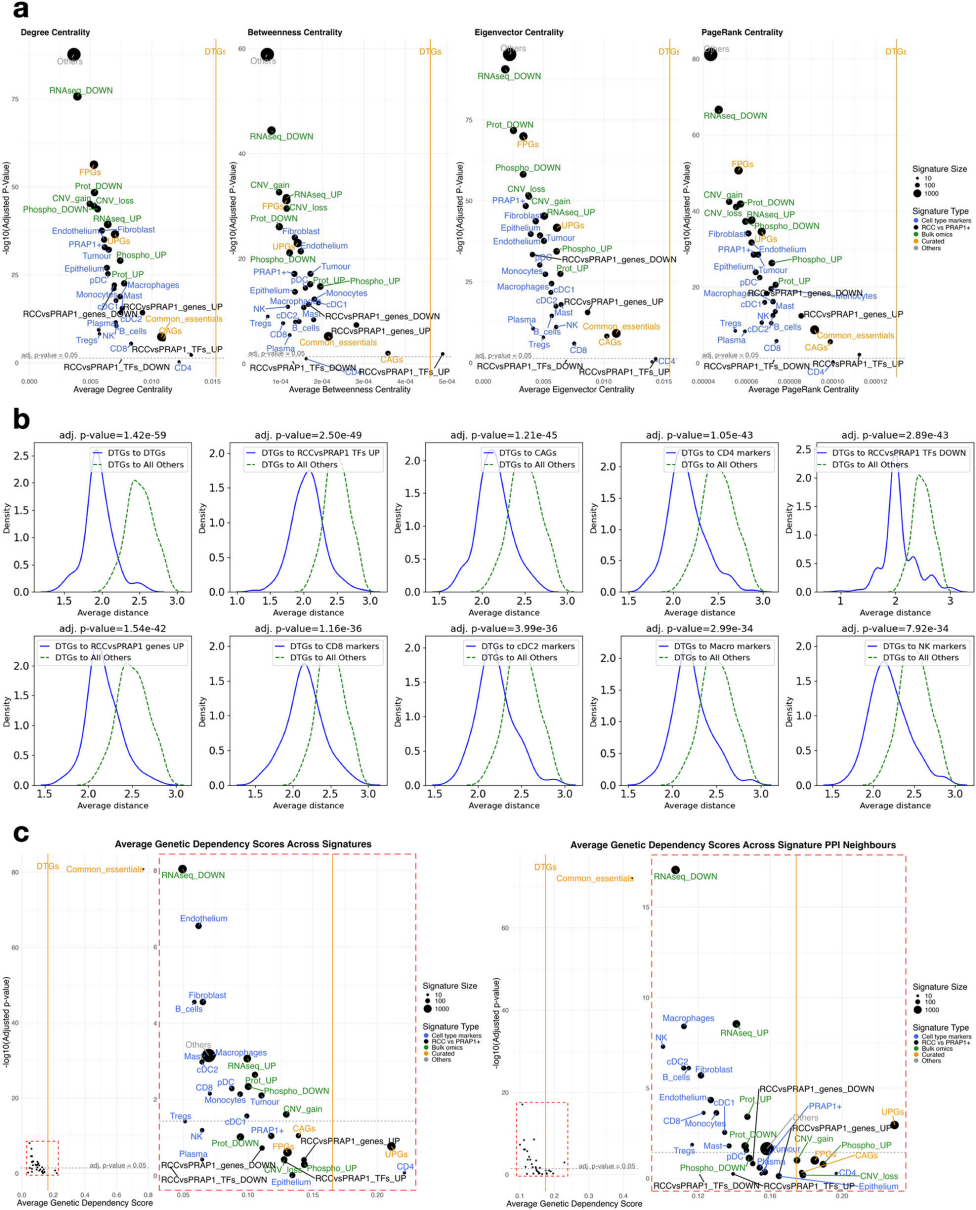
Drug target gene and candidate disease-associated gene signature characteristics. **a** Relationship between average network centrality and statistical significance of centrality differences relative to DTGs. The *x*-axis shows the average centrality score (degree, betweenness, eigenvector, or PageRank) of each signature within the human protein–protein interaction (PPI) network. The *y*-axis represents the statistical significance of the difference in centrality between each CDAG set and the DTG set, expressed as –log₁₀ of the BH-adjusted *p*-value (Wilcoxon rank-sum test). **b** Comparison of average shortest-path distance distributions within the PPI network, measuring the proximity of DTGs to a given signature (blue) versus to all remaining network nodes (green). The top 10 most significantly different distributions are shown. Statistical significance was assessed using BH-adjusted Wilcoxon rank-sum tests; adjusted *p*-values are indicated. **c** Average genetic dependency of CDAG signatures relative to DTGs, based on CRISPR knockout screens across 16 ccRCC cell lines (DepMap data). The left panel shows results for genes within each signature; the right plot shows results for the immediate PPI neighbours of each signature. The *x*-axis indicates the average dependency scores, and the *y*-axis corresponds to –log₁₀ of the BH-adjusted *p*-value for dependency differences (Wilcoxon rank-sum test).

Recognising that genes within biological systems reveal their functions when viewed in relation to one another, we mapped the DTG and CDAG signatures onto the STRING human protein–protein interaction network (PPI; STRING^38^ database, confidence threshold ≥400; 19,303 proteins), previously shown to be among the best-performing molecular networks for disease gene discovery^39^. Using this network, we first interrogated average DTG and CDAG centralities (Fig. 3a). This analysis revealed that DTGs exhibit the highest average degree, eigenvector, and PageRank centralities, underscoring their roles as hub nodes. Next, we characterised the DTG set in relation to the compiled 35 signatures by comparing the average shortest-path distances within the PPI network (Fig. 3b, Supplementary Fig. 4). Specifically, we calculated the average shortest paths from each gene in the DTG set to every gene in a given signature and compared them to the average shortest paths from each DTG to all other nodes in the network not part of that signature. Overall, DTGs were found to be closer to the curated gene sets than to the rest of the gene population, with the exception of down-regulated bulk RNA-seq transcripts, genes with copy number gains, and those classified under the ‘Others’ category.

We also investigated gene essentiality across 16 ccRCC cell lines using systematic CRISPR knockout data from the DepMap^20^ database, focusing on 17,880 proteins shared between the STRING PPI network and DepMap data. Dependency scores ranging near 1 signify a gene’s criticality for cell survival – its knockout leading to marked growth inhibition or cell death. Conversely, scores near 0 imply negligible impact on cell growth upon gene knockout. Notably, dependency data was not available for three DTGs (*CSF2RA*, *IL3RA*, and *XRCC7*). A comparative analysis of the average gene dependency scores between the remaining 199 DTGs and CDAGs revealed that only one gene set, the common essential genes, exhibited significantly higher dependency scores. (Fig. 3c). This observation is expected, given that common essential genes are defined as the most consistently depleted across multiple cell lines. The lack of significantly higher average dependency in any other gene set indicates that DTGs tend to cause more pronounced growth arrest in ccRCC cell lines. However, it is important to note that DTGs are not universally essential for ccRCC lines, as genes are considered essential only if their dependency score exceeds 0.5, whereas the average for DTGs is ~0.17. This observation aligns with the fact that most ccRCC therapeutics directly affect the tumour microenvironment rather than the tumour cells themselves, as seen with VEGF pathway inhibitors and immunotherapies. A similar trend was observed when comparing the average dependency scores of each gene set’s PPI neighbours, with the common essential gene neighbourhood again showing the highest average dependency.

Overall, the outlined results confirm that the curated signatures were not merely random selections of genes but are functionally relevant to the disease. Namely, the relatively high DTG network centralities indicate their involvement in multiple pathways, which, when disrupted, lead to disease regression. This finding is further supported by the comparatively high genetic dependency scores associated with DTGs. Finally, the close proximity among DTGs suggests that they form a network module, while their proximity to CDAGs indicates that CDAGs are involved in pathways essential for cancer maintenance and development.

### Machine learning model construction and performance

**Fig. 4:**
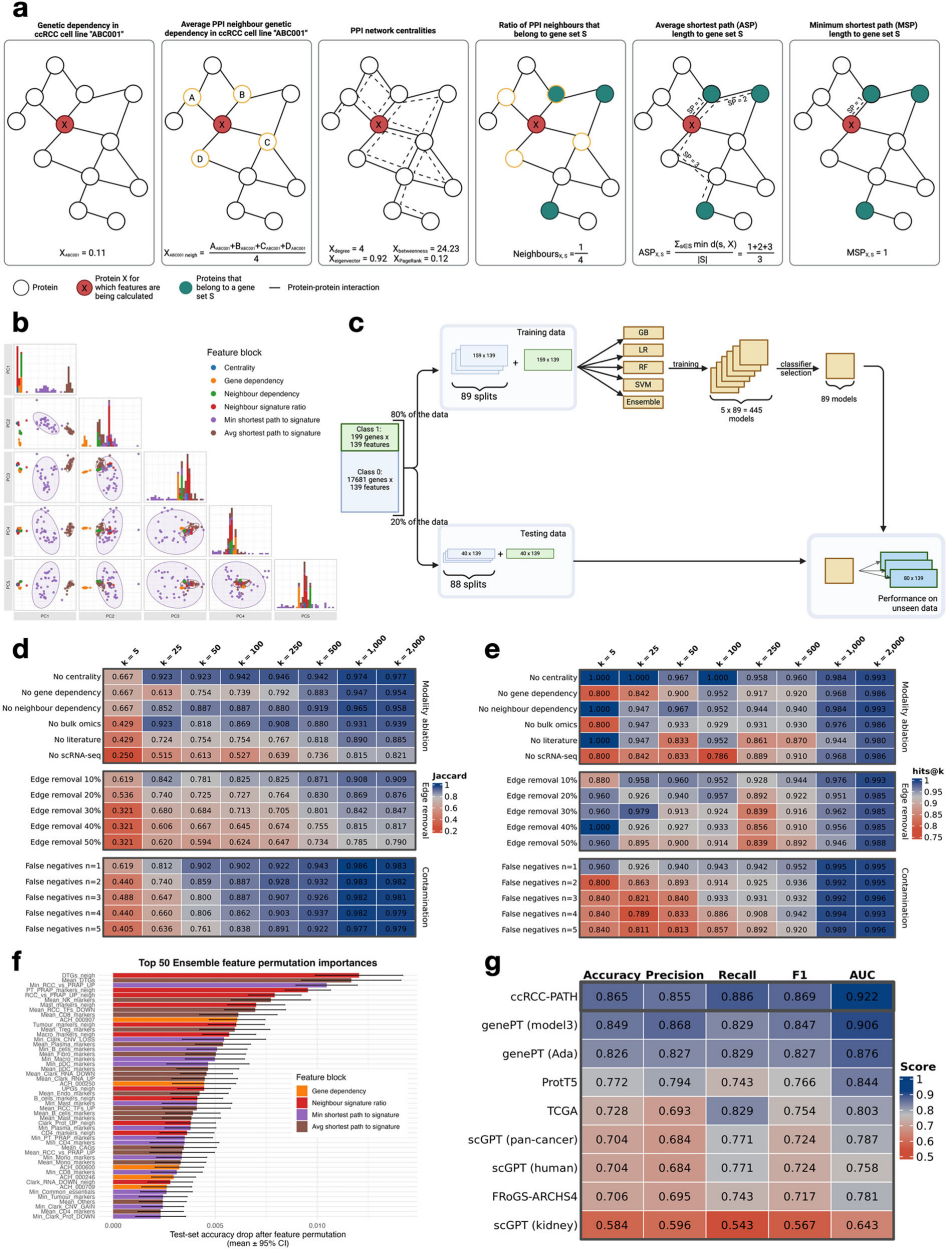
Machine learning for the identification of novel therapeutic targets for ccRCC. **a** Six feature type blocks used for gene characterisation. Genetic dependency scores across 16 ccRCC cell lines (16 features), along with average dependency scores of each gene’s PPI neighbours in the same cell lines (16 features); PPI network centralities (degree, betweenness, eigenvector, PageRank; 4 features); and features derived from 35 curated disease signatures, including neighbour proportions (35 features), average shortest-path distances (35 features), and minimum shortest-path distances (33 features). **b** Feature organisation across leading principal components. Pairwise projections of the first five principal components of the 139-dimensional gene embedding, where each point represents a feature, coloured by feature block. **c** Machine learning workflow. Protein-coding genes were assigned to Class 1 (ccRCC drug targets) or Class 0 (all other genes) and split 80/20 for model training and testing. To ensure balanced representation, Class 0 genes were randomly sampled exactly once to match the count of Class 1 genes. Each resulting balanced dataset was used to train four machine learning classifiers and their ensemble. Classifier performance was assessed via 5-fold CV, and the best-performing method was subsequently evaluated on the held-out test set and used for final genome-wide predictions. **d** Stability of top-k predictions under model perturbations. Jaccard similarity of top-k ranked genes relative to the baseline model across modality ablation, label contamination, and PPI edge removal analyses. **e** Relative recovery of known targets (Class 1 genes) among top-k predictions under model perturbations. Hits@k relative to the baseline model across the same perturbation analyses as in (**d**). **f** Permutation-based feature importance of the ensemble classifier on the held-out test set, shown for the top 50 features ranked by mean decrease in test accuracy. Bars indicate mean ±95% CI across 100 permutations. **g** Benchmarking ccRCC-PATH. Test set performance of ccRCC-PATH and pre-trained gene embeddings.

Our findings align with the work of Isik et al.^11^, who demonstrated that the proximity between drug targets and genes deregulated by the same drug yields the highest target prediction accuracy. Building on this insight, we extended and generalised the approach by leveraging 35 disease signatures to compute 139 features for each of the 17,880 genes, capturing both their genetic dependency profiles and their positioning within the PPI network (Supplementary Data 4). Therefore, instead of predicting targets of a particular compound based on its perturbation profile, such gene-level characterisation allows us to predict the so-called targets of the disease given multiple disease signatures. These 139 features were subdivided into six blocks (Fig. 4a): genetic dependency scores across 16 ccRCC cell lines (16 features); average genetic dependency scores of a gene’s PPI neighbours across the same 16 ccRCC cell lines (16 features); PPI network centralities^1^ (4 features); neighbourhood composition^40^ - defined as the fraction of a gene’s PPI neighbours that belong to a given signature (35 features); average shortest-path distance to a signature^11^ - defined as the mean shortest-path length from a gene to all genes in that signature (35 features); and finally, minimum shortest-path distance to a signature^7^ - defined as the shortest-path length from a gene to the nearest member of that signature (33 features; minimum distances to DTGs and genes belonging to the ‘Others’ set were excluded to avoid label leakage and prevent biasing predictions towards already known gene-disease associations). To assess relationships among the constructed features, we next performed principal component analysis (PCA) of the resulting gene embedding (Fig. 4b). PC1-PC2 and pairwise PC1-PC5 projections showed clear clustering of features by their predefined blocks, with PC1 contrasting distance-to-signature features (brown and purple clusters) with local neighbourhood composition and centrality metrics (red and blue clusters), while PC2 separated minimum-distance proximity features (purple cluster) from CRISPR genetic dependency signals (orange and green clusters) (Supplementary Fig. 5). Higher-order components captured additional, biologically coherent structure within the feature space, including contrasts between tumour/epithelial and immune-lineage proximity patterns, rather than diffuse or unstructured feature mixing.

Building on the work by Tsagkogeorga et al.^41^, we next designated genes encoding DTGs as the positive class (199/17,880; Class 1) and all remaining genes as the negative class (17,681/17,880; Class 0), with the aim of developing a machine learning predictor capable of distinguishing these two classes of genes using the 139 features. We partitioned these classes in an 80/20 split for model training and testing (Fig. 4c). To ensure balanced datasets, negative-class genes in both training and testing sets were randomly sampled exactly once to match the number of Class 1 genes. Subsequently, four machine learning classifiers (Logistic Regression (LR), Support Vector Machine (SVM), Random Forest (RF), and Gradient Boosting (GB)) and their ensemble (combining LR, SVM, RF, and GB) were trained using the 89 balanced training datasets. Classifiers showed strong concordance in class probability distributions and top-ranked predictions (Supplementary Fig. 6), while 5-fold cross-validation results (Table 2) identified the ensemble classifier as the best-performing method across all metrics except precision.

**Table 2:** performance metrics across 5 ML classifiers obtained using 5-fold CV on the training data

Metric	Ensemble	GB	LR	RF	SVM
Accuracy	0.8475 ± 0.0298	0.8461 ± 0.0340	0.8313 ± 0.0352	0.8409 ± 0.0363	0.8393 ± 0.0326
Precision	0.8361 ± 0.0361	0.8370 ± 0.0427	0.8189 ± 0.0428	0.8326 ± 0.0426	0.8257 ± 0.0406
Recall	0.8688 ± 0.0341	0.8647 ± 0.0408	0.8558 ± 0.0342	0.8581 ± 0.0466	0.8650 ± 0.0546
F1	0.8504 ± 0.0283	0.8487 ± 0.0327	0.8350 ± 0.0330	0.8432 ± 0.0359	0.8429 ± 0.0332
AUC	0.9173 ± 0.0250	0.9147 ± 0.0264	0.8993 ± 0.0312	0.9151 ± 0.0258	0.9068 ± 0.0287

Next, we systematically assessed the robustness of the resulting baseline model using four perturbation analyses: feature filtering, leave-one-modality-out ablation, false-negative label contamination, and progressive PPI edge removal (Fig. 4d, e, Supplementary Figs. 7–10). To evaluate whether the relatively high feature dimensionality could inflate model performance through redundancy or over-parameterisation, we applied univariate feature filtering by removing 2.5–30% of features ranked lowest by variance, F-statistic, or mutual information with the class label, followed by model retraining. Across all three filtering criteria, performance remained stable after removal of up to ~20% of features, whereas more aggressive filtering (≥25–30%) led to a consistent decline across metrics (Supplementary Fig. 7). Importantly, none of the filtering strategies outperformed the full 139-feature embedding, arguing against overfitting due to excessive feature redundancy and indicating that predictive signal is not concentrated in a small subset of dominant features. Next, to quantify the contribution of individual data layers, we performed leave-one-modality-out ablations across network centrality, gene and neighbour CRISPR dependency, literature-derived, bulk-omics, and scRNA-seq features. Removal of scRNA-seq, bulk-omics, or literature-derived features produced the largest changes in performance, with scRNA-seq ablation most strongly reducing recall and F1 score, and literature feature removal yielding the largest AUC decrease (Supplementary Fig. 8). In contrast, ablation of CRISPR gene dependency features caused only minor changes in ML performance metrics but led to noticeable reordering of top-ranked candidates (Fig. 4d, e). Namely, early recovery of known targets (Class 1 genes) was reduced, while broader rankings at *k* = 1000 showed minimal changes in both hits@k and Jaccard overlap. Ranking stability was most strongly affected by scRNA-seq data removal (Jaccard@25 ≈ 0.52; Jaccard@1000 ≈ 0.82; hits@25 ≈ 0.84, hits@1000 ≈ 0.97); literature- and bulk-omics ablations showed intermediate effects; and removal of centrality features had minimal impact.

Because gene labels are inherently imperfect, reflecting the evolving therapeutic target landscape, we assessed robustness to false negatives using controlled label contamination. Using the same 89 balanced datasets, we randomly flipped 1–5 positive (Class 1) labels to the negative class (Class 0) within cross-validation training folds, repeating this procedure across five random seeds per contamination level (Supplementary Fig. 9). Increasing contamination led to a decrease in recall accompanied by increase in precision, consistent with a more conservative classifier under noisy positive labels, while AUC and accuracy remained largely unchanged. Ranking-based analyses showed that contamination primarily affected early prioritisation, with relative hits@25 decreasing to ~0.81 and Jaccard@25 to ~0.64 at the highest contamination level, whereas broader ranking structure was largely preserved (hits@1000 ≈ 0.99, Jaccard@1000 ≈ 0.98; Fig. 4d, e). In a complementary robustness analysis, we evaluated sensitivity to PPI network incompleteness by randomly removing 10–50% of edges from the STRING interactome, generating five independently perturbed networks per removal level and recomputing all PPI-dependent features prior to retraining. Increasing network sparsity led to gradual degradation of classification performance, most prominently affecting recall, while overall discrimination remained comparatively robust (Supplementary Fig. 10). At the level of target prioritisation, edge removal induced progressive rank reshuffling, particularly at small k: Jaccard@25 decreased from ~0.84 at 10% removal to ~0.62 at 50% removal, whereas Jaccard@1000 remained substantially higher (~0.91–0.79; Fig. 4d, e). Despite this rank reshuffling, recovery of known targets remained high (hits@25 ≈ 0.90, hits@1000 ≈ 0.95 at 50% edge removal), demonstrating the framework’s resilience under substantial network degradation.

We next evaluated the ensemble classifier on held-out test data (40 DTGs and 3530 negative-class genes), achieving an accuracy of 0.8569, precision of 0.8335, recall of 0.900, F1 score of 0.8644, and AUC of 0.9254. Averaging Class 1 probabilities across the 89 ensemble models, 660 of 3530 negative-class genes exceeded a probability threshold of 0.5, corresponding to a false-positive rate of 0.187. Finally, permutation-based feature importance analysis on the held-out test data showed that network proximity and neighbourhood enrichment features produced the largest decreases in test accuracy upon permutation, while no single-feature dominated model behaviour (Fig. 4f). Together with the feature filtering and ablation results, these findings indicate that predictive performance arises from the cumulative integration of multiple partially redundant yet complementary signals, rather than reliance on a small number of dominant features. Consistent with this, perturbation analyses show that the overall target prioritisation ordering is preserved under label noise and network sparsification, with sensitivity confined primarily to fine-grained early ranks.

We next benchmarked ccRCC-PATH against eight recently published gene embeddings derived from biomedical literature (genePT model3, genePT Ada^42^), single-cell transcriptomics (scGPT pan-cancer, scGPT human, scGPT kidney^43^), bulk transcriptomics (TCGA^44^, FRoGS-ARCHS4^45^), and protein sequence data (protT5^46^). Restricting the analysis to the 15,002 genes shared across all embeddings, we used a common train/test split and evaluated all representations within the same classification framework (Fig. 4g; Supplementary Fig. 11). Across both 5-fold cross-validation on the training data and evaluation on the unseen test split, the ccRCC embedding consistently achieved the strongest overall performance among all tested models. The literature-based genePT model3 approached comparable discrimination to that of ccRCC-PATH, albeit with a more conservative prediction profile characterised by higher precision and reduced recall.

### New therapeutic target prediction

By ranking previously uncharacterised genes in ccRCC, the ccRCC-PATH framework yielded a comprehensive resource of novel disease effectors for experimental validation and therapeutic development (Supplementary Data 5). To narrow down top predictions obtained from ccRCC-PATH, we utilised AstraZeneca’s 5R’s R&D^47^ framework (right target, right patient, right tissue, right safety, right commercial potential). For the ‘Right target’ and ‘Right tissue’ criteria, we focused on the top 10 novel predictions that directly impact tumour cells. This selection process considered genes markedly over-expressed in bulk proteomic data or, if such data were unavailable, transcriptomic data from tumour versus normal tissue comparisons (log2FC > 0.5, adj. *p*-value < 0.05). Candidate genes were further required to be over-expressed in tumour cells relative to all other broad cell types in scRNA-seq data (log2FC > 0.25, adj. *p*-value < 0.05), as well as relative to the PT PRAP1+ epithelial cluster (log2FC > 0.5, adj. *p*-value < 0.05). In addition, genes were filtered for high cell-type specificity, defined by a scRNA-seq Tau score greater than 0.5 as reported by the Human Protein Atlas. The top 10 candidates that fit the criteria were: Lysyl oxidase (*LOX*), Enolase 2 (*ENO2*), Leucine rich repeat kinase 2 (*LRRK2*), Transglutaminase 2 (*TGM2*), Haem oxygenase 1 (*HMOX1*), Scavenger receptor class B member 1 (*SCARB1*), Caveolin-1 (*CAV1*), Transforming growth factor alpha (*TGFA*), c-Myc (*MYC*), and Intercellular Adhesion Molecule 1 (*ICAM1*).

**Fig. 5:**
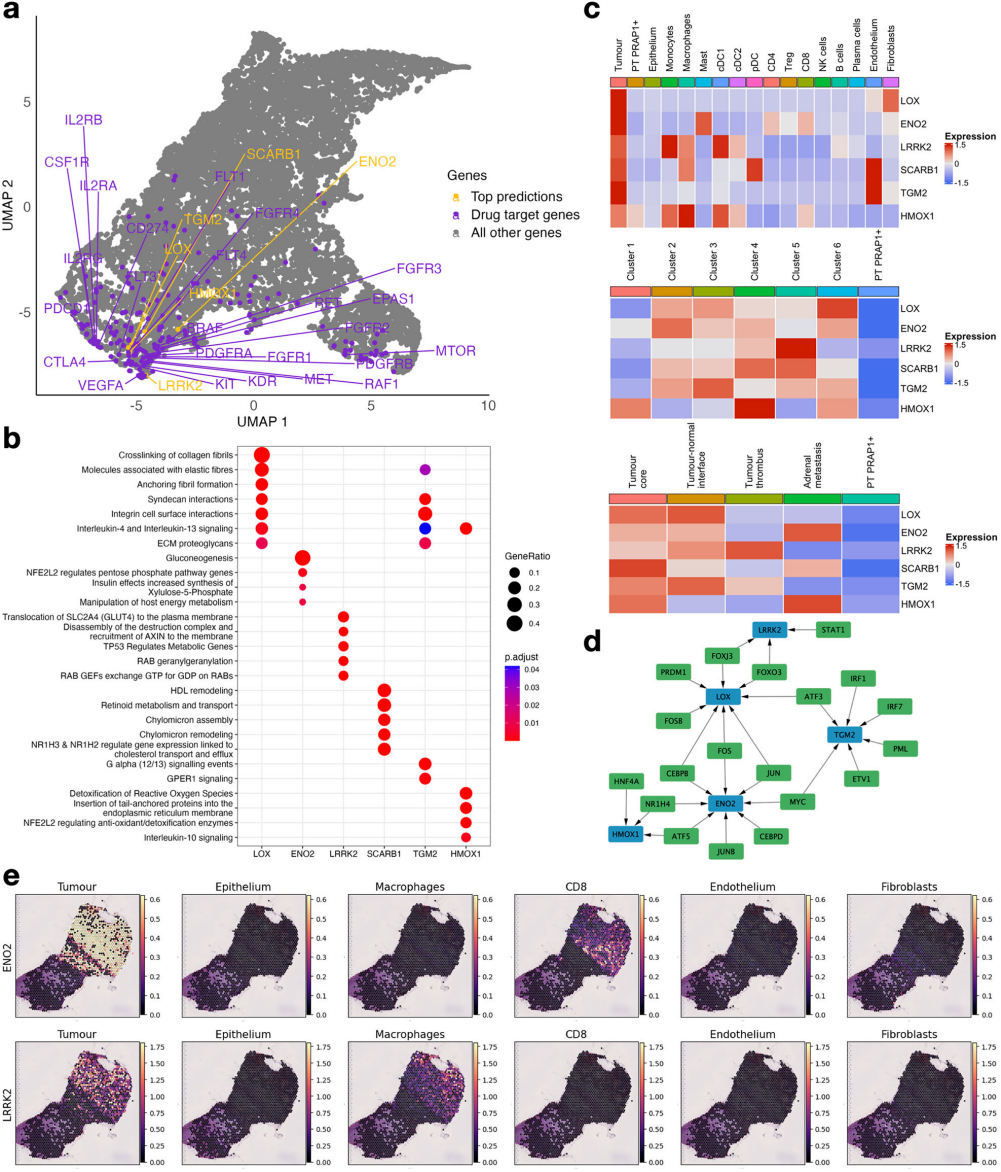
Characterisation of top tumour target predictions. **a** UMAP visualisation of the feature space used to train and evaluate the machine learning target prediction framework, comprising 17,880 genes and 139 features. Genes belonging to the drug target gene (DTG) set are highlighted in purple. Within this group, genes targeted by FDA-approved therapies for ccRCC are highlighted and labelled in purple, while those currently under investigation in clinical trials are highlighted in purple without labels. The refined set of top tumour target predictions is highlighted in yellow and labelled. **b** Reactome pathway enrichment analysis of the protein–protein interaction (PPI) neighbourhoods of the six top tumour target predictions. High-confidence interaction partners (confidence score ≥700) were retrieved from the STRING database to define each candidate’s PPI neighbourhood. Reactome pathway enrichment was performed on these neighbourhoods, and results were filtered to retain only pathways corresponding to terminal nodes (leaf pathways) in the Reactome hierarchy, ensuring specificity of functional annotations. **c** scRNA-seq expression profiles of the six top candidate genes. Top: expression levels across broad cell types. Middle: expression patterns across tumour and proximal tubule PRAP1⁺ (PT PRAP1⁺) cell clusters. Bottom: expression comparison between tumour and PT PRAP1⁺ cells, stratified by the anatomical region sampled for sequencing. **d** Inferred transcriptional regulators of the six predicted targets in tumour cells, identified using the SCENIC pipeline. **e** ENO2 and LRRK2 expression patterns across indicated cell types at the tumour-normal interface in spatial transcriptomics data from ccRCC patient PD47171.

For ‘Right safety,’ genes identified as common essentials by DepMap^20^ CRISPR or RNAi projects, namely *MYC*, were excluded. The ‘Right patient’ criterion was met by focusing exclusively on ccRCC data, and the ‘Right commercial’ aspect was supported by the high prevalence of kidney cancer and the unmet clinical need in patients who experience primary progression despite current standard-of-care therapies. The remaining nine genes were further narrowed down by removing those without available small-molecule inhibitors, resulting in the exclusion of *CAV1* and *TGFA*. Finally, we also excluded immune-related predictions – *ICAM1* – in order to permit validation of our hypotheses in a non-immune experimental model. Visualisation of the ML feature space revealed a highly cohesive clustering of known and newly identified targets (Fig. 5a). This, in turn, further demonstrates that the 139 features used to characterise each gene capture the biological information that determines whether that gene is a likely ccRCC therapeutic target.

To elucidate the functions of the remaining predicted targets, Reactome^37^ pathway enrichment analysis was conducted for the high-confidence PPI neighbourhoods of these genes (Fig. 5b; confidence score ≥700). The results revealed that *LOX* and *TGM2* are involved in extracellular matrix organisation, *ENO2* is associated with glucose metabolism, *LRRK2* participates in multiple pathways, including *GLUT4* translocation and membrane trafficking, *HMOX1* is involved in the inactivation of reactive oxygen species, and *SCARB1* plays a role in lipid metabolism. Regarding target scRNA-seq expression profiles, all six genes demonstrated good tumour specificity, with the additional cell types expressing some of these genes being myeloid and endothelial cells (Fig. 5c). Next, SCENIC^23^ output was analysed to identify target transcriptional regulators. *SCARB1* had no predicted TFs regulating its expression, likely due to the stringent filtering criteria applied (see “Methods” section). The remaining five genes, along with their predicted TFs, formed a connected network, as depicted in Fig. 5d. Finally, spatial transcriptomics data of the ccRCC tumour core and the tumour-normal interface were evaluated. The results corroborated scRNA-seq findings, confirming that the six target genes are specific to tumour rather than the adjacent normal tissues (Fig. 5e; Supplementary Fig. 12).

### Experimental validation of predicted targets

**Fig. 6:**
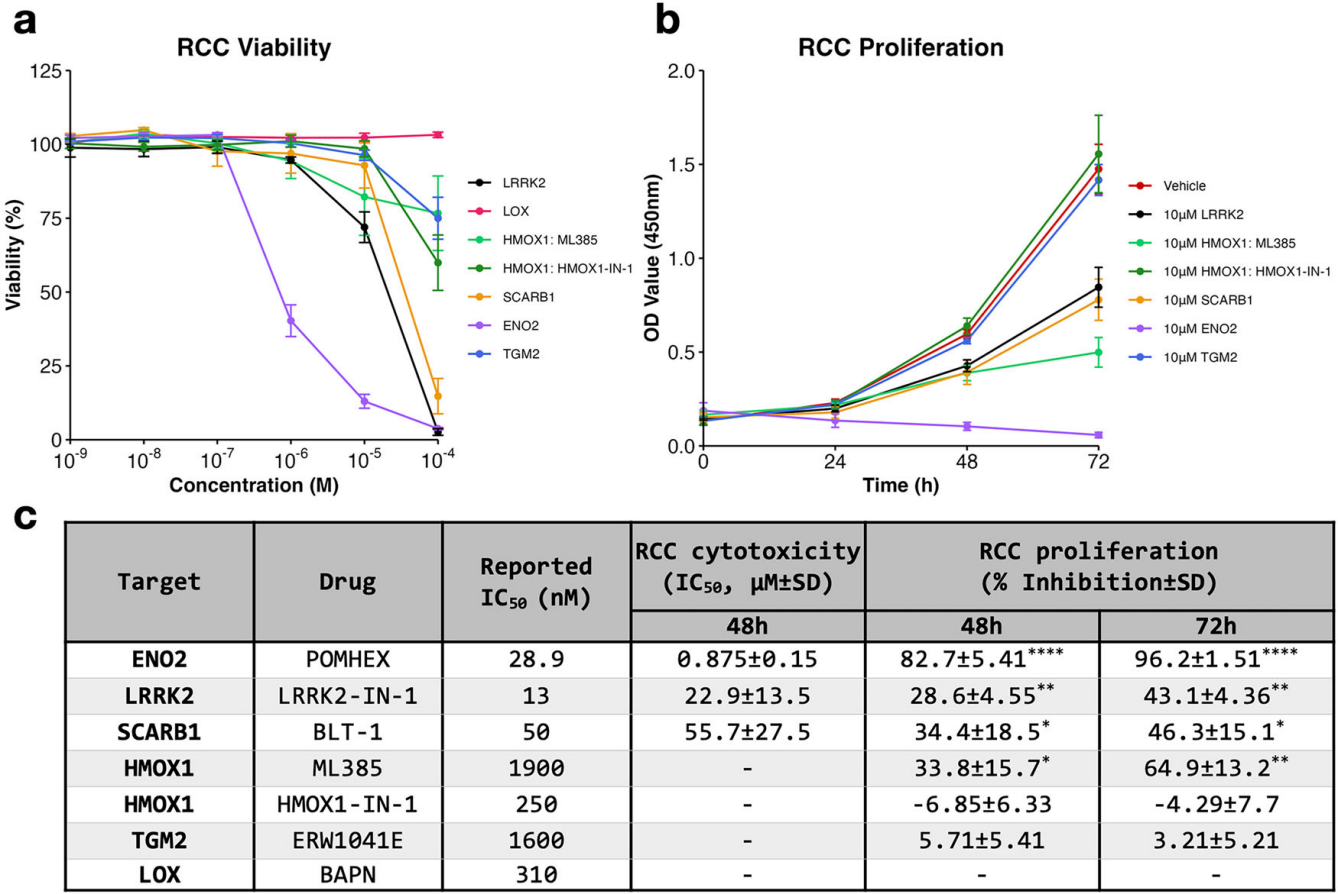
Experimental validation of top tumour target predictions. **a** Cytotoxicity assay results. Three ccRCC cell lines were treated in triplicate for 48 h with either vehicle controls or inhibitors targeting LRRK2, LOX, HMOX1 (via its upstream regulator NFE2L2 using ML385 or directly using HMOX1-IN-1), SCARB1, ENO2, and TGM2, across six concentrations (1 nM, 10 nM, 100 nM, 1 µM, 10 µM, 100 µM). Cell viability was calculated relative to DMSO (10^−^^5^ or 10^−^^4^M; LRRK2, HMOX1, SCARB1, ENO2, TGM2) or ddH_2_O (10^−^^4^M; LOX) treated controls after background subtraction. For each cell line, technical replicates were averaged, and the resulting cell line means were used to calculate the overall mean ± SEM across the three cell lines. **b** Proliferation inhibition assay results. Three ccRCC cell lines were treated in triplicate for 72 h with either vehicle controls or 10 µM of inhibitors targeting the genes of interest. Background-corrected absorbance values are reported as mean ± SEM across three cell line averages. **c** Summary of drug response in RCC. Data are presented as mean ± SD across three cell line averages. Asterisks indicate significance compared to vehicle control (Kruskal–Wallis, Dunn’s multiple comparison; *P < 0.05, **P < 0.01, ****P < 0.0001). Dashes indicate conditions that were not tested or for which values could not be determined.

The six predicted targets were subsequently evaluated in three ccRCC cell lines using small-molecule inhibitors. Cytotoxicity assays revealed that inhibition of *ENO2* (POMHEX^48^, IC_50_ = 28.9 nM) *LRRK2* (LRRK2-IN-1^49^, IC_50_ = 13 nM), and *SCARB1* (BLT-1^50^, IC_50_ = 50 nM) produced the most pronounced reduction in cell viability, with the corresponding average inhibitor cytotoxicity IC_50_ values across the tested cell lines being 0.875, 22.9, and 55.7 μM (Fig. 6a, c; Supplementary Fig. 13). This was further supported by proliferation assays (Fig. 6b**;** Supplementary Fig. 14), where treatment with 10 μM of the respective inhibitors led to an average reduction in cell proliferation of approximately 45% for *SCARB1* and *LRRK2*, and nearly complete inhibition for *ENO2*.

For *HMOX1*, two drugs were tested: one directly targeting the gene (HMOX1-IN-1^51^, IC_50_ = 0.25 μM), and one inhibiting the upstream regulator *NFE2L2*^52^ (ML385^53^, IC_50_ = 1.9 μM). Along with *TGM2* suppression (ERW1041E^54^, IC_50_ = 1.6 μM), inhibitor effects were cell line-specific: 786-O cells were most sensitive to ML385 and ERW1041E, whereas A498 cells showed the greatest sensitivity to HMOX1-IN-1. The comparatively weaker response to *TGM2* and *HMOX1* inhibition, relative to *ENO2* or *LRRK2*, is likely attributable to the higher IC_50_ values of the respective inhibitors. Higher compound concentrations were not considered due to DMSO-associated cytotoxicity above 10^−^^4^M (Supplementary Fig. 15). In contrast, targeting *LOX* (β-Aminopropionitrile^55^, IC_50_ = 0.31 μM) did not affect RCC cell viability.

### Generalisability of the PATH framework across diseases

Finally, to assess the generalisability of PATH, we applied our model to lung adenocarcinoma (LUAD), representing an independent disease context. To construct LUAD-PATH, we identified 240 DTGs, of which 236 had available CRISPR dependency data (Supplementary Data 6). CDAGs were derived from a previously published LUAD scRNA-seq dataset^56^ by identifying differentially expressed genes across 22 cell types (Supplementary Fig. 16; Supplementary Table 1, Supplementary Data 7), and were complemented with bulk tumour-normal signatures from RNA-seq, proteomics, phosphoproteomics, acetylproteomics, and whole-genome sequencing copy number data from the Gillette et al. cohort^57^. Additional LUAD-related gene sets were obtained from DisGeNET^18^, the Human Protein Atlas^19^, and DepMap^20^ databases. Together, these data yielded a LUAD-specific gene embedding comprising 246 features (Supplementary Data 8). The same train/test splitting and balanced training procedure used for ccRCC-PATH was then applied, generating 75 balanced datasets for model training. As in the ccRCC analysis, the ensemble classifier achieved the strongest performance during five-fold cross-validation on the training data (accuracy >0.8; AUC > 0.9; Supplementary Table 2) and was therefore evaluated on the held-out test set, where it achieved an accuracy of 0.849, precision of 0.837, recall of 0.872, F1 score of 0.854, and AUC of 0.923, demonstrating that the framework can successfully recapitulate known drug targets in an independent cancer type (Supplementary Fig. 17; Supplementary Data 9).

## Discussion

In this study, we have presented a multimodal framework that integrates disease-associated gene signatures, genome-wide CRISPR knockout data, and PPI network features to systematically prioritise therapeutic targets. Applied to clear-cell renal cell carcinoma, this approach identified five candidate genes, among which *ENO2* and *LRRK2* demonstrated the most potent anti-ccRCC effects, highlighting their potential as targets for therapeutic intervention.

The key strength of the framework lies in its capacity to integrate data from multiple modalities, providing a flexible and scalable approach that can be readily extended to other diseases. By mapping disease signatures onto the human interactome and computing both average and minimum shortest-path distances to each gene, we quantify how readily a particular node can communicate with the signatures. These topological features are further complemented by local PPI neighbourhood information and CRISPR knockout data, generating a feature space that characterises each gene with respect to the disease of interest. Crucially, this approach does not rely on known functional associations such as pathway data, thereby mitigating biases toward well-characterised biological processes. These features are then fed into a machine learning pipeline that addresses class imbalance and uses standard classifiers to discriminate known drug targets (Class 1 genes) from the broader gene pool. Beyond target prioritisation, the flexibility of the framework also allows it to be adapted to other biological questions – such as gene function inference – provided an appropriate positive class can be defined. Consistent with this generalisability, application of PATH to LUAD using an independently constructed multimodal embedding reproduced strong predictive performance and recovery of known targets, demonstrating that the approach extends beyond ccRCC.

Another key advantage of the approach is its ability to anchor predictions in empirical evidence. By training on known drug targets, the model learns not only from topological context but also from functional perturbation outcomes, providing a more biologically informed alternative to unsupervised approaches that rank genes solely based on individual topological features such as centrality or proximity^7,12^. For example, while DTGs exhibited higher centrality and closer proximity to disease signatures, permutation-based feature importance results revealed that no single feature was dominating. This observation is further supported by perturbation analyses, which showed that removing up to ~20% of features based on variance or univariate association did not enhance performance, while more aggressive filtering consistently reduced it. Together, these results indicated that predictive performance emerged from the cumulative integration of multiple complementary signals rather than reliance on a small set of dominant predictors. Consistent with this integrative design, benchmarking against modern pre-trained biological embeddings showed that the disease-specific representation achieved stronger overall discrimination within the same classification framework, supporting the effectiveness of our approach for disease-specific target prioritisation.

While the framework demonstrates strong predictive performance, several limitations should be acknowledged. First, although it effectively prioritises candidate targets, it does not provide mechanistic insights into how or why targeting a particular gene may confer therapeutic benefit. As such, downstream experimental validation remains essential to establish causal relevance. Second, the approach relies on protein–protein interaction networks, which remain incomplete and subject to biases arising from uneven research attention across genes. However, random edge removal analyses showed that recovery of known targets remained high even under substantial network sparsification, indicating resilience to realistic levels of network incompleteness. Third, the classification of genes into positive (known targets) and negative (non-targets) classes is inherently imperfect. In the context of ccRCC, many of the curated drugs remain under clinical investigation, and some genes labelled as non-targets may, in fact, represent emerging or as-yet-unvalidated therapeutic candidates. Controlled label contamination experiments showed that increasing false negatives primarily affected early target prioritisation while leaving global discrimination largely intact, supporting the model’s ability to tolerate label noise. Consistent with this, the model’s high accuracy (0.8569) and AUC (0.9254) on unseen data underscore its practical utility for target prioritisation despite residual data incompleteness and label uncertainty.

Given the limited availability of drugs that directly target ccRCC tumour cells rather than the tumour microenvironment, we focused on top-ranked candidates with tumour cell specificity. Functional validation across three ccRCC cell lines demonstrated that inhibition of *ENO2* (POMHEX, IC_50_ = 28.9 nM), *LRRK2* (LRRK2-IN-1, IC_50_ = 13 nM) and *SCARB1* (BLT-1, IC_50_ = 50 nM) produced the most potent and consistent effects. These results align with prior studies reporting that knockdown of either *ENO2*^58^ or *SCARB1*^59^ significantly suppresses ccRCC growth both in vitro and in vivo. Mechanistically, *ENO2* knockdown induces ferroptosis - a nonapoptotic, iron- and lipid-dependent form of cell death^60^. Ferroptosis represents a targetable vulnerability in ccRCC, with prior studies demonstrating that *HIF-2α* is the central driver of this vulnerability through selective enrichment of polyunsaturated lipids, the rate-limiting substrates for lipid peroxidation^61,62^. Similarly, *SCARB1* is also heavily involved in lipid metabolism, with ccRCC relying on *SCARB1* for high-density lipoprotein (HDL) import. Genome-wide association studies (GWAS) have identified a single nucleotide polymorphism at the *SCARB1* locus associated with increased ccRCC risk, with Mendelian randomisation analyses of GWAS data further supporting a causal relationship between genetic alleles linked to elevated circulating HDL levels and ccRCC occurrence^63,64^.

For *LRRK2*, while its role has primarily been studied in the context of neurodegeneration – with its inhibitor BIIB122 currently undergoing phase III clinical trials for Parkinson’s disease – emerging evidence supports its relevance in ccRCC. Yang et al.^65^ identified *LRRK2* as a putative prognostic biomarker in ccRCC, reporting that aberrant *LRRK2* expression is associated with altered DNA methylation patterns and that *LRRK2* knockdown significantly reduces A498 and 786-O cell line proliferation. In parallel, GWAS results have linked *LRRK2* to elevated blood cholesterol levels^66,67^. Based on these findings, and prior to the publication of Hong et al.^68^, we selected *LRRK2* for further investigation, as its role in ccRCC had, to our knowledge, been explored only once previously - in the study by Yang et al.^65^. Hong et al.^68^ independently demonstrated that *LRRK2* knockdown in vivo significantly slowed ccRCC tumour progression and that *LRRK2* promotes resistance to tyrosine kinase inhibitors and immunotherapy by stabilising a lipid metabolism gene *LPCAT1*. We note that this study was published after our model predictions had been finalised, highlighting that our machine learning framework can uncover clinically meaningful therapeutic targets independently of prior experimental reports.

In contrast, responses to *HMOX1* and *TGM2* inhibition were more variable and appeared to be cell line-dependent, although the comparatively lower potency of their respective inhibitors limits direct comparison with the other compounds. Previous studies have shown that *HGF-MET* signalling induces the Ras-Raf-ERK pathway, which, in turn, activates *HMOX1* to counteract oxidative stress. Consistently, ccRCC cells treated with *HGF* show increased *NFE2L2* nuclear translocation compared with vehicle-treated controls^69^. Furthermore, inhibition of *HMOX1*, either alone or in combination with *MET*, has been reported to reduce ccRCC tumour volume, decrease tumour vessel density, and increase oxidative stress in vivo^70^. Finally, *HMOX1* downregulation is also known to cause ccRCC mitotic delay at the G2/M phase^71^. Our findings support these observations, with *NFE2L2* inhibition resulting in an average proliferation reduction of 65% across the three cell lines. Meanwhile, *TGM2* blockade has been shown to induce apoptosis in ccRCC mouse xenograft models through *p53* stabilisation^72–74^. The resulting elevated *p53* levels were also found to reduce angiogenesis by decreasing HIF-1α activity through the promotion of the *p300–p53* interaction and the concurrent reduction in the *p300–HIF-1α* interaction^75^.

In summary, this study presents a novel computational framework for systematic therapeutic target discovery, integrating disease signatures, functional genomics, and PPI network analysis within a machine learning pipeline. The identification of multiple ccRCC-relevant candidates, including *ENO2* and *LRRK2*, highlights the effectiveness of the approach in uncovering functionally meaningful targets. The framework’s flexibility and scalability allow it to be applied to other diseases, providing a structured approach to target prioritisation in diverse biological contexts.

## Methods

### scRNA-seq data acquisition and pre-processing

The scRNA-seq dataset was downloaded from Mendeley Data (https://data.mendeley.com/datasets/g67bkbnhhg/1). The data was subset to exclude two patients (PD44714 and PD47172) who had malignancies other than ccRCC.

### Broad cell-type identification

The filtered Seurat^76^ object was split according to the patient of origin using the *SplitObject* function, followed by normalisation of UMI counts (*NormalizeData*) and identification of 2000 most variable features (*FindVariableFeatures*) for each patient independently. Next, features that exhibited repeated variation across patients were determined using the *SelectIntegrationFeatures* function, and principal component analysis (PCA) was performed separately for each patient using these features. Integration anchors were subsequently calculated using the *FindIntegrationAnchors* function, setting the reduction parameter to ‘rpca’, followed by dataset integration (*IntegrateData*). Finally, the standard workflow involving data scaling (*ScaleData*), PCA calculation (*RunPCA*), UMAP drawing (*RunUMAP*), nearest-neighbour graph construction (*FindNeighbors*), and cluster determination (*FindClusters*) was carried out. Differentially expressed genes among the broad cell types were determined using the *FindAllMarkers* function; DEGs with log2FC > 1 and adjusted p-value < 0.05 were used to define the broad cell-type marker gene lists.

### TF activity inference

The level of TF activity was estimated using pySCENIC^23^ (v.0.9.15; Python implementation of SCENIC). Due to the exceptionally large number of identified cells, it was not computationally feasible to perform single-cell regulatory network calculation for the entire dataset in a single run. To circumvent this challenge, the dataset was split according to the identified broad cell types. Subsequently, each cell subset was individually filtered to exclude transcripts that occurred in fewer than 5 cells. *hg38__refseq-r80__10kb_up_and_down_tss.mc9nr.feather* and *hg38__refseq-r80__500bp_up_and_100bp_down_tss.mc9nr.feather* databases, as well as the human v9 motif collection, were obtained from cisTarget (https://resources.aertslab.org/cistarget/). The GRNBoost2 algorithm was implemented for gene regulatory network inference, followed by the computation of enriched motifs and regulon predictions using the CLI ctx function. Finally, to estimate regulon activity scores in each cell, the aucell function was utilised. In the analysis of tumour cells, the workflow had one modification: SCENIC GRN inference was concurrently conducted for both tumour and PT PRAP1+ epithelial cells. This modification facilitated a direct comparison of TF activities between these two cell populations. Regulons demonstrating an absolute log2FC greater than 0.5 and an adjusted p-value below 0.05 were classified into activated and inactivated TF signatures.

### Data clustering and annotation

For each broad cell type, a total of 20 independent pySCENIC runs were performed. Results were subsequently filtered so that only regulons detected in a minimum of 19 out of 20 runs were retained. Meanwhile, the targets of the remaining TFs were filtered by selecting those that recurred in 19/19 or 20/20 runs. For each broad cell-type subset, the resulting 20 filtered AUCell matrices were averaged to produce a single AUCell matrix, which was subsequently used to create a Seurat^76^ object. Batch correction was performed using Seurat’s integration pipeline. Integration anchors were determined using the *FindIntegrationAnchors* function with default parameters and using all of the remaining regulons as features. An integrated Seurat object was obtained using the *IntegrateData* function with default parameters as well. Data was then scaled (*ScaleData*), and PCA performed (*RunPCA*), using all of the filtered regulons as features. Next, nearest-neighbour graph construction, cluster determination, and non-linear dimensionality reduction were carried out using *FindNeighbors*, *FindClusters* and *RunUMAP* functions, respectively. Differentially active regulons were identified using the *FindAllMarkers* function. The resulting cluster labels were then transferred to the original scRNA-seq Seurat object of the respective broad cell type, followed by scRNA-seq UMI count normalisation (*SCTransform*) and differential gene expression analysis (*FindAllMarkers*). Regarding tumour vs PT PRAP1+ differential expression analysis results, genes with an absolute log2FC value above 1 and an adjusted p-value below 0.05 were classified into up- and down-regulated signatures.

### Tumour meta-programme determination and characterisation

The integrated Seurat^76^ tumour cell object containing regulon activity scores (see above) was split according to the patient of origin. Next, for each patient, the corresponding AUCell matrix was scaled using the *ScaleData* function, followed by replacing all negative values in the matrix with zero. For each tumour, the top 10 regulon activity modules were calculated using the *nmf* function of the NMF package (v0.26). For each regulon module, the top ten regulons with the highest weight were selected to define an intra-tumour activity programme. The resulting regulon modules were then filtered to retain only those that had standard deviations above 0.2 among tumour cells. Finally, the resulting intra-tumour regulon activity modules were clustered based on pairwise Jaccard index

$${Jaccard}\,{index}=\,\frac{A\cap B}{A\cup B}$$

where A and B correspond to two intra-tumour regulon activity programmes. This allowed the identification of regulon meta-programmes that are shared across multiple tumours. Regulons shared by at least 50% of tumours with a particular meta-programme were used to define that meta-programme. Seurat *AddModuleScore* function with ‘nbin’ and ‘ctrl’ parameters being 6 and 50, respectively, was used to calculate the average activity levels of each meta-programme in each tumour cell. Reactome^37^ pathway enrichment analysis of the meta-programmes was carried out using the standard workflow of the gprofiler2^77^ package.

### Collection of prognostic, ccRCC-associated, common essential, and drug target genes

Prognostic genes were retrieved from the Human Protein Atlas^19^ (HPA) using the search term “Renal cancer”. The resulting list was further split into favourable and unfavourable prognosis gene signatures (FPGs and UPGs, respectively) based on survival analysis. ccRCC-associated genes (CAGs) were obtained from DisGeNET^18^ using the following search terms: “Conventional (Clear Cell) Renal Cell Carcinoma”, “Clear-cell metastatic renal cell carcinoma”, and “Hereditary clear-cell renal cell carcinoma”; only curated associations were considered. Common essential genes from the CRISPR and RNAi screens were sourced from the DepMap^20^ database; the union of these two lists was used as a signature in downstream analyses. Clinical trial data was downloaded from the DrugBank^78^ database, focusing on trials investigating potential treatments (instead of, for example, diagnostic, preventative, or supportive care). Furthermore, trials that were withdrawn, terminated, or suspended were excluded. Separately, only those target genes that have a ‘yes’ for DrugBank’s pharmacological action section were included in the Class 1 set to ensure that the drug directly interacts with the target. For drug entries missing from the database or annotated as stubs, a literature search was conducted to identify their targets.

### Pre-processing and differential abundance testing of bulk-omics data

Bulk ccRCC omics data from 110 donors were sourced from the Clark et al.^79^ study. Across all omics layers, non-ccRCC samples (C3L-00359, C3N-00313, C3N-00435, C3N-00492, C3N-00832, C3N-01175, and C3N-01180) and their corresponding normal adjacent tissue samples, as identified by the original authors, were excluded from the analyses.

Bulk RNA-seq data were acquired using the TCGAbiolinks^80^ package. During pre-processing, a contaminated transcriptomics sample (C3N-00314) was also excluded. Count data were filtered to retain transcripts with at least 10 counts in ≥50% of healthy or tumour samples. Library size normalisation factors were then computed using the “TMM” method (*calcNormFactors*), and expression values were transformed with the *voom* function. For donors with multiple sequencing runs of the same tissue type (healthy or tumour), expression values were averaged. Differentially expressed genes were identified using the *lmFit* and *eBayes* functions of the limma^81^ package. Genes with an absolute log2FC value above 2 and an adjusted p-value below 0.05 were used to define up- and down-regulated transcript signatures.

Copy number variation data were also obtained using the TCGAbiolinks^80^ package. To identify genes with recurrent CNVs, we first excluded those located on the X and Y chromosomes. For donors with multiple sequencing runs, CNV values were averaged. Next, genes with missing values in more than 50% of samples were removed. Following guidelines from the Catalogue of Somatic Mutations in Cancer (https://cancer.sanger.ac.uk/cosmic/help/cnv/overview), a gene was defined as amplified if present in ≥5 copies and as deleted if completely absent in a given donor. CNV frequencies were then calculated across all samples, and the top 1000 genes most frequently affected by gains or losses were retained. This corresponded to a minimum of 7 donors for deletions and 26 donors for amplifications.

Proteomics and phosphoproteomics data were obtained from the CPTAC data portal (https://cptac-data-portal.georgetown.edu/cptac/s/S050; CPTAC3_CCRCC_Whole_abundance_gene_protNorm =2_CB.tsv; 6_CPTAC3_CCRCC_Phospho_abundance_phosphopeptide_protNorm=2_CB_imputed_1211.tsv). For proteomics data, proteins with more than 50% of missing values were removed, while the remaining missing values were imputed using the DreamAI ensemble method (https://github.com/WangLab-MSSM/DreamAI). Differential protein and phosphopeptide abundance analyses were conducted utilising the *lmFit* and *eBayes* functions of the limma^81^ package. Significant changes in protein levels in tumour tissues were identified with criteria of an absolute log2FC greater than 1 and an adjusted p-value below 0.05. For phosphoproteomics, a gene was considered differentially phosphorylated if at least one of its phosphosites had an absolute log2FC greater than 1 and an adjusted *p*-value below 0.05.

### Gene embedding construction

Human protein–protein interaction (PPI) data were sourced from the STRING^38^ (v11.5) database and filtered to retain interactions with a combined confidence score of ≥400. Using the networkx^82^ package, we first computed degree, betweenness, eigenvector, and PageRank centralities for each node within the network. Next, we mapped the 35 gene signatures (Table 1) onto the PPI network and derived gene-level graph descriptors for each gene based on its relationship to each signature. Specifically, we computed (i) the minimum and (ii) average shortest-path distance from a given gene to all genes in a signature, and (iii) the relative neighbourhood composition, defined as the fraction of a gene’s direct PPI neighbours that belong to a signature. Notably, minimum-distance features to DTG and ‘Others’ signatures were excluded to prevent label leakage and avoid biasing predictions towards genes with a minimum distance to ‘Others’ greater than 0, respectively.

CRISPR gene dependency data were obtained from the DepMap^20^ database, followed by filtering to retain all available (*n* = 16) ccRCC cell lines. Missing dependency values for a given gene in a specific cell line were imputed using the mean dependency score across cell lines with non-missing values for that gene. Average gene dependency scores of all PPI neighbours of a particular gene were calculated for each cell line individually.

### Machine learning methodology: framework, model optimisation, and performance evaluation

To estimate the therapeutic potential of individual genes, we applied conventional machine learning methods for binary classification. The 199 known drug target genes (DTGs) were assigned as the positive class (Class 1) and split into a training and cross-validation set (80%, *n* = 159) and an independent test set (20%, *n* = 40) held out for final model evaluation. The negative class (Class 0) comprised the remaining gene pool (*n* = 17,681), which was similarly divided into training (80%, *n* = 14,151) and test (80%, *n* = 3530) sets. Here, the assumption was that most genes fulfil roles other than as therapeutic targets, thereby minimising the incidence of false negatives within the training data. To create balanced training sets and reduce model variance through averaging, the 14,151 negative training samples were partitioned into 89 non-overlapping subsets of 159 genes each, matching the number of positive training samples. Therefore, each balanced training set consisted of the same 159 positive samples and a unique set of 159 negative samples, ensuring every negative gene was sampled exactly once across all training iterations.

Subsequently, we applied five machine learning classifiers: Logistic Regression (LR), Support Vector Machine (SVM), Random Forest (RF), Gradient Boosting (GB), and an ensemble model that averaged the predicted Class 1 probabilities across LR, SVM, RF, and GB methods. For SVM, hyperparameter tuning was conducted using grid search with 5-fold cross-validation, optimising the kernel function (linear or RBF), cost parameter, and kernel bandwidth (for the RBF kernel). For RF, an initial grid search determined the optimal forest size, followed by a randomised search to fine-tune the number of features considered for node splitting, maximum tree depth, and minimum sample thresholds for node splits and leaf nodes. Similarly, the GB model’s learning rate and forest size were optimised via grid search, with a subsequent randomised search for decision tree parameters (analogous to RF). All five models were trained on each of the 89 balanced training sets. Performance was assessed using 5-fold cross-validation based on standard classification metrics: accuracy, precision, recall, F1 score, and area under the receiver operating characteristic curve (AUROC).

### Model perturbation analyses

To assess whether reducing the feature space could improve model generalisation, univariate feature filtering was performed using variance, F-statistic, or mutual information with the class label. Feature scores were computed on the training data across the 89 balanced datasets and aggregated using the mean to obtain a single consensus score per feature. Quantile-based thresholds were then applied to remove the bottom 2.5–30% of features, and all downstream analyses were performed using these fixed filtered feature sets.

To quantify the contribution of each data modality, we derived a series of leave-one-modality-out embeddings from the 139-feature baseline representation. Specifically, we constructed embeddings excluding: (i) network centrality features (4 features); (ii) CRISPR gene dependency features (16); (iii) CRISPR neighbour dependency features (16); (iv) literature-derived features (removal of features derived from the DTG signature, common essential gene signature, favourable and unfavourable prognostic gene signatures, and the ccRCC-associated gene signature; 14); (v) bulk multi-omics features derived from Clark et al.^79^ data (24); and (vi) scRNA-seq-derived features (63). For ablations involving gene signature features (DTGs or CDAGs), the corresponding ‘Others’ signature features were re-computed using the reduced signature sets. For each ablated embedding, balanced training datasets were reconstructed using the same set of genes as in the baseline model, and classifiers were retrained using the identical classification procedure applied to the full 139-feature embedding.

To evaluate robustness to false negatives, we conducted a label contamination analysis. Using the same 89 balanced datasets as in the baseline model, controlled label noise was introduced by randomly flipping 1–5 positive-class (Class 1) gene labels to Class 0 within the training folds only of a 5-fold cross-validation procedure; validation folds remained clean. Model performance was assessed using accuracy, precision, recall, F1 score, and AUC. For each balanced dataset, the final classifier was obtained by retraining on the full balanced dataset with the same contamination level. To quantify variability arising from stochastic label noise, the entire procedure was repeated five times for each contamination level (1–5 flipped genes), yielding five independent contamination models per level, each generated using a distinct random seed assignment across the 89 balanced datasets.

To examine robustness to incomplete protein–protein interaction knowledge, we performed random edge removal on the STRING v11.5 network (confidence threshold ≥400). To preserve network connectivity after sparsification, a spanning tree of the graph was computed, and edges belonging to the spanning tree were excluded from removal. A specified fraction of the remaining (non-tree) edges was then randomly sampled and removed. For each removal level (10%, 20%, 30%, 40%, or 50%), five independently randomised pruned networks were generated and used to recompute network-derived features. For each resulting embedding, balanced training datasets were reconstructed using the same set of genes as in the baseline model, and classifiers were retrained using the identical classification procedure applied to the full 139-feature embedding.

### Model testing on unseen data and feature importance analysis

Upon establishing the ensemble classifier as the best-performing method based on cross-validation, we proceeded to evaluate its performance and feature importance on unseen data. Mirroring the approach employed for the training procedure, we generated 88 balanced test sets. This involved partitioning the 3530 negative-class genes reserved for testing into groups of 40 – matching the number of held-out positive samples. Each negative group was paired with the same set of 40 positive test samples to form the test sets. Each of the 89 ensemble models was then evaluated on all 88 test sets using accuracy, precision, recall, F1 score, and AUROC metrics. Final performance was calculated by averaging results across all ensemble models and test sets. Feature importance was assessed by independently permuting each feature through random shuffling of its values across genes while keeping all other features unchanged, followed by recomputation of test accuracy. This procedure was repeated 100 times per feature for each balanced test set, and feature importance was quantified as the average decrease in accuracy relative to the unpermuted baseline.

### Benchmarking of ccRCC-PATH

GenePT^42^, scGPT^43^, TCGA^44^, and FRoGS-ARCHS4^45^ embeddings were obtained from their respective publicly available repositories, while the protT5^46^ embedding was retrieved from the STRING^83^ database. Together with the ccRCC-PATH embedding, all representations were pre-processed by mapping gene identifiers to Entrez IDs, followed by identification of the set of genes shared across all embeddings (15,002 genes; Class 1 = 177, Class 0 = 14,825). Each embedding was then restricted to this common gene set, and a shared train/test split was constructed. For each embedding, training data (Class 1 = 142; Class 0 = 11,928) were partitioned into 84 balanced training datasets using identical gene sets, followed by model training under the same classification framework. Performance was subsequently evaluated on the held-out test data (Class 1 = 35, Class 0 = 2897) using 83 test sets.

### Spatial transcriptomics data analysis

Tumour core and tumour-normal interface spatial transcriptomics data was obtained from Mendeley Data (https://data.mendeley.com/datasets/g67bkbnhhg/1). The Cell2location^84^ package was implemented to map cell clusters from scRNA-seq data to the 10X Genomics Visium spatial transcriptomics measurements. First, basic filtering of spots was carried out using Scanpy’s^85^ *pp.filter cells* function to remove spots with transcript counts below 2000 or above 35,000, less than 500 detected genes, and a mitochondrial gene fraction exceeding 20%. Additionally, slides 6800STDY12499504 and 6800STDY12499505 were also removed due to having fewer than 500 spots. Next, scRNA-seq data was filtered using Cell2location’s *filter_genes* function with the following parameters: ‘cell_count_cutoff’ = 5, ‘cell_percentage_cutoff2’ = 0.01, ‘nonz_mean_cutoff’ = 1.05. The unnormalised mRNA count matrix was used as input for this filtering step, after which 16,285 genes and 250,331 cells remained in the dataset. Next, a negative binomial regression model was implemented to estimate the reference signature of cell types detected in the scRNA-seq dataset. Here, patient IDs were used as batch keys, and the model was trained using the *mod.train* function with the following parameters: ‘max_epochs’ = 100, ‘batch_size’ = 2500, ‘train_size’ = 1, ‘lr’ = 0.002. The summary of the posterior distribution was exported using the *mod.export_posterior* function. The resulting reference signature model was then used to predict the spatial abundance of cell types. Only genes identified in both scRNA-seq and spatial transcriptomics were retained. Cell-type abundance was estimated separately for the tumour core and tumour-normal interface slides, employing the following parameters: ‘N_cells_per_location’ = 20, ‘detection_alpha’ = 200, ‘max_epochs’ = 30,000.

### In vitro experiments

#### Cell lines

A498, 769-P and 786-O cell lines were kindly provided by the Sakari Vanharanta laboratory. Cells were expanded using R10 medium (RPMI1640 (Gibco), 10% Fetal Bovine Serum (Sigma), 1% Penicillin-Streptomycin (Sigma) and 2 mM L-Glutamine (Gibco)). Cells were expanded using adherent T75s in 37 °C, 5% CO_2_ incubators with Accutase (Invitrogen) passaging. All cells used for experiments were below passage 15.

#### Drugs

ML385 (SML1833-5MG), BLT-1 (373210-25MG), POMHEX (HY-131904-5MG), ERW1041E (5095220001), LRRK2-IN-1 (Biotechne Tocris, #4273), and HMOX1-IN-1 (TA9H94532D16-5MG) powders were solubilised in DMSO (Invitrogen). BAPN (S5340-25MG) was solubilised in ddH_2_O. All reconstituted solutions were stored as directed by the manufacturer.

#### Cytotoxicity

5000 proliferating cells in 25 µL of R10 medium were added to wells of pre-warmed adherent 384-well plates. After 24 h at 37 °C, 5% CO_2_, R10 medium was aspirated before 25 µL drug-supplemented R10 medium or DMSO controls were added. Supplemented-R10 was incubated with cells for 2 days, before 25 µL CellTiterGlo3D Viability Assay (ProMega) administration and viability determination through luminescence recording by FLUOstar Optima (BMG Labtech). Drugs were assessed across two experimental rounds: round 1 examined ML385, BLT-1, POMHEX, ERW1041E, and BAPN, while round 2 examined LRRK2-IN-1 and HMOX1-IN-1.

#### Proliferation

4000 or 2500 proliferating cells (A498/769-P or 786-O) were seeded per 96 well plate well in 100 µL of drug or control supplemented R10 medium. Plates were equilibrated for 2 h at 37 °C, 5% CO_2_, before 10 µL CCK8 (abcam) was added with incubation for 4 h, 37 °C, 5% CO_2_ and the recording of absorbance at 450 nm using the FLUOstar Optima (BMG Labtech). CCK8 testing was repeated on separate plates on days 1, 2 and 3, with drug-supplemented medium-only controls included for each time point to account for background CCK8 reduction. Drugs were assessed across two experimental rounds: round 1 examined ML385, BLT-1, POMHEX, ERW1041E, and BAPN, while round 2 examined LRRK2-IN-1 and HMOX1-IN-1. For plotting purposes, to account for minor differences in baseline vehicle proliferation between experimental rounds, OD450 values from round 2 were rescaled such that the mean background-corrected vehicle OD450 per cell line and time point matched the corresponding values from round 1.

#### Statistical analyses

All data are presented as the mean ± standard error of the mean (SEM) or standard deviation (SD). Statistical analyses and IC_50_ value calculations were performed in GraphPad Prism 10.2.3. For comparisons involving more than three groups, statistical significance was determined using the Kruskal–Wallis test followed by Dunn’s multiple comparisons test.

### LUAD-PATH embedding construction and evaluation

To assess framework generalisability, we constructed a LUAD-specific PATH embedding using the same methodology as for ccRCC. LUAD Class 1 genes, favourable and unfavourable prognostic genes, and LUAD-associated genes were curated from DrugBank^78^, the Human Protein Atlas^19^ (query term: “lung adenocarcinoma”), and DisGeNET^18^ (query term: “adenocarcinoma of lung”). The common essential gene signature was identical to that used in ccRCC-PATH. CRISPR gene dependency data across 53 LUAD cell lines were obtained from the DepMap^20^ database and pre-processed identically to the ccRCC cell lines. Bulk multi-omics LUAD signatures were derived from RNA-seq, proteomics, phosphoproteomics, acetylproteomics, and whole-genome sequencing tumour-normal analyses reported by Gillette et al.^57^, applying the same log2FC and statistical significance thresholds as in the ccRCC analyses.

To generate scRNA-seq – derived features, we analysed the GSE131907 dataset^56^, retaining lung-derived cells (“nLung”, “tLung”, “tL/B”) with high-confidence cell-type annotations provided by the original authors. Differential expression across 22 cell types and tumour-epithelial comparisons (AT1, AT2, ciliated, and club cells) was performed using the same log2FC and statistical significance thresholds as in the ccRCC analyses.

All features were integrated to construct a 246-dimensional LUAD embedding, which was split into training and test sets using an 80/20 partition. The training data (Class 1 = 189; Class 0 = 14,175) were used to generate 75 balanced sets and train the same classification framework as for ccRCC. Based on a 5-fold CV, the ensemble classifier was selected and evaluated on held-out test data (Class 1 = 47; Class 0 = 3469) using 74 test sets.

## Supplementary information

Supplementary_information_reformatted_v1

Supplementary Data 1

Supplementary Data 2

Supplementary Data 3

Supplementary Data 4

Supplementary Data 5

Supplementary Data 6

Supplementary Data 7

Supplementary Data 8

Supplementary Data 9

nr-reporting-summary_v1

## Data Availability

The ccRCC scRNA-seq and spatial transcriptomics datasets are available from Mendeley Data (https://data.mendeley.com/datasets/g67bkbnhhg/1). Bulk ccRCC omics data are available from the CPTAC data portal (https://cptac-data-portal.georgetown.edu/cptac/s/S050). CRISPR gene dependency data are available from the DepMap portal (https://depmap.org; 23Q4 release). The LUAD scRNA-seq dataset is available from GEO (GSE131907).

## Code availability

The code for embedding generation and model training is made available at 10.5281/zenodo.19240170.
