# Supplementary material for: A network medicine framework for multi-modal data integration in therapeutic target discovery
Source: Commun Chem. 2026 May 6;9:244. doi: 10.1038/s42004-026-02049-9 (PMC13358115; doi:10.1038/s42004-026-02049-9)
Supplement: Supplementary file 1 — Supplementary_information_reformatted_v1 [file 42004_2026_2049_MOESM1_ESM.pdf]

Supplementary information

A network medicine framework for multi-modal data integration in therapeutic target discovery

Greta Baltušytė[1,2,3,4], Isaac Toleman[2], James O. Jones[5,6], Sarah J. Welsh[2,6], Grant D. Stewart[2,6], Thomas J. Mitchell[2,6,7], Kourosh Saeb-Parsy[2,*,†], Namshik Han[1,3,4,8,9,10*,†]

1 Milner Therapeutics Institute, University of Cambridge, Cambridge, UK

2 Department of Surgery, University of Cambridge, and Cambridge NIHR Biomedical Research Centre, Cambridge, UK

3 Cambridge Stem Cell Institute, University of Cambridge, Cambridge, UK

4 Cambridge Centre for AI in Medicine, University of Cambridge, Cambridge, UK

5 Department of Oncology, University of Cambridge, Cambridge, UK

6 Cambridge University Hospitals NHS Foundation Trust, Cambridge, UK

7 Wellcome Trust Sanger Institute, Wellcome Genome Campus, Cambridge, UK

8 Department of Quantum Information, Institute for Convergence Research and Education in Advanced Technology and Engineering, Yonsei University, Seoul, Republic of Korea

9 Department of Nano Biomedical Engineering (NanoBME), Advanced Science Institute, Yonsei University, Seoul, Republic of Korea

10 Center for Nanomedicine, Institute for Basic Science (IBS), Seoul, Republic of Korea

* Corresponding authors: ks10014@cam.ac.uk, nh417@cam.ac.uk

† These authors jointly supervised this work

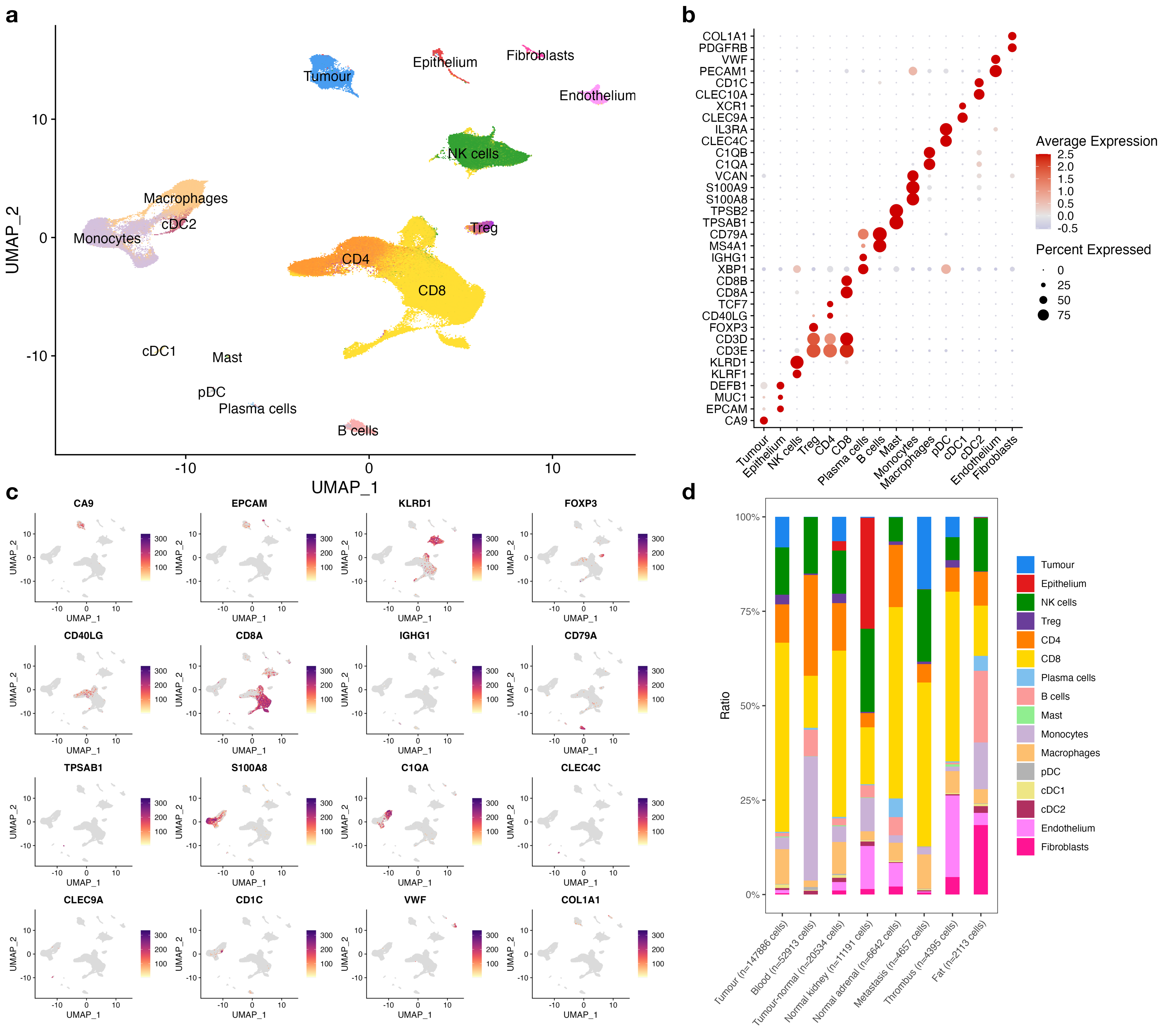

Supplementary Figure 1: Single-cell transcriptomic profiling of ccRCC reveals 16 broad cell compartments.

a Uniform manifold approximation and projection (UMAP) visualisation of 250,331 cells obtained from 10 histopathologically confirmed ccRCC donors in the Li et al. cohort. Cells are coloured by the 16 broad cell compartments identified through unsupervised clustering and canonical marker gene expression. Multiple anatomical sites were sampled from each donor, including the tumour core, tumour-normal interface, adjacent normal kidney, peripheral blood, perinephric fat, normal adrenal gland, adrenal metastasis, and tumour thrombus, where available. **b** Expression of canonical cell type marker genes used to annotate the 16 cell compartments. **c** UMAP visualisation from (**a**), overlaid with the expression of representative marker genes used to define major cellular compartments. **d** Bar plot showing the distribution of the 250,331 profiled cells across eight anatomical regions sampled for sequencing.

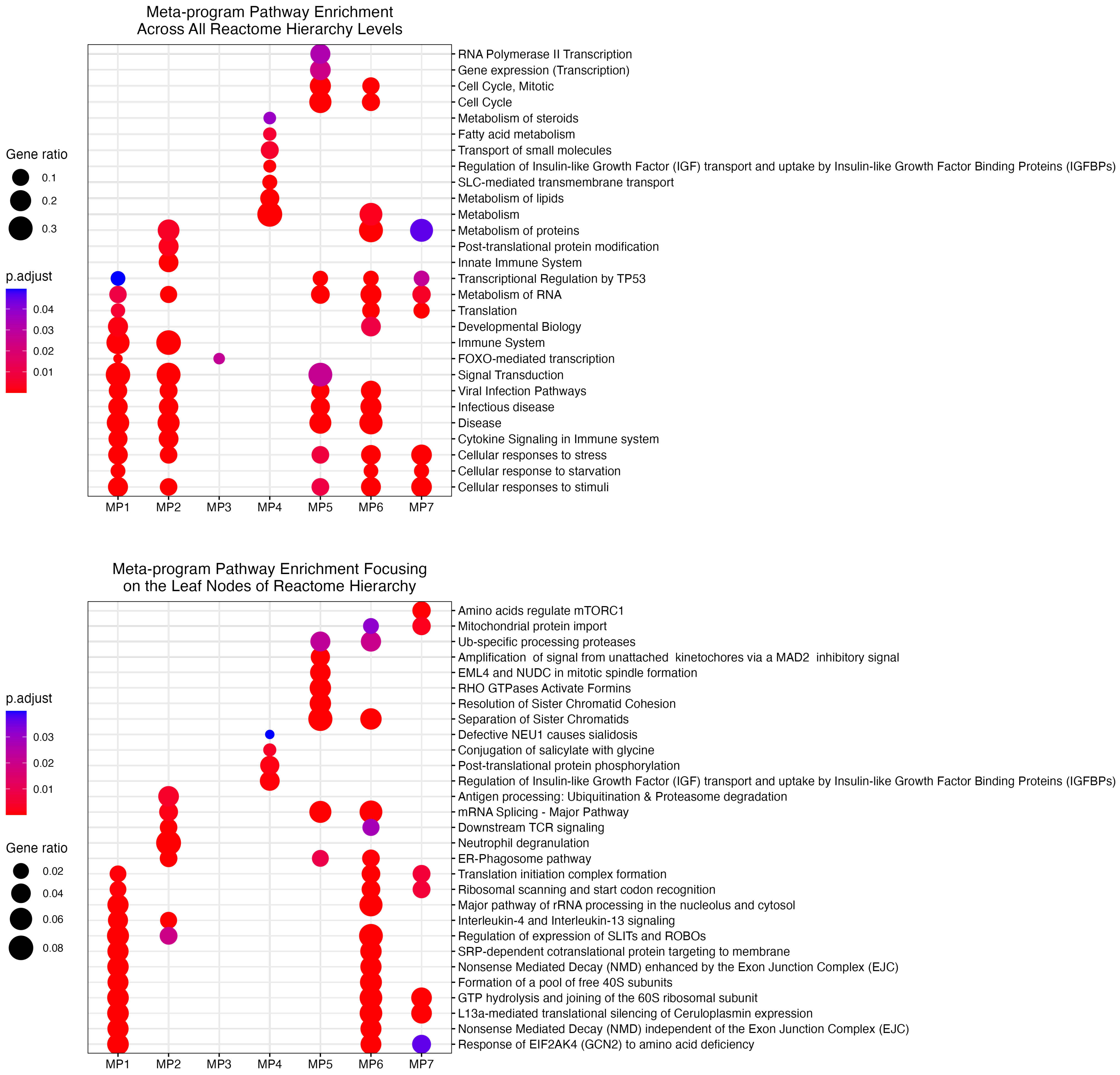

Supplementary Figure 2: Reactome pathway enrichment analysis of tumour meta-programs. Transcription factors and their target genes defining each meta-program were used to perform Reactome pathway enrichment analyses. Top panel: analysis results incorporating pathways at all levels of the Reactome pathway hierarchy. Bottom panel: enrichment analysis focused exclusively on the terminal (leaf) nodes of the hierarchy, ensuring specificity of functional annotations.

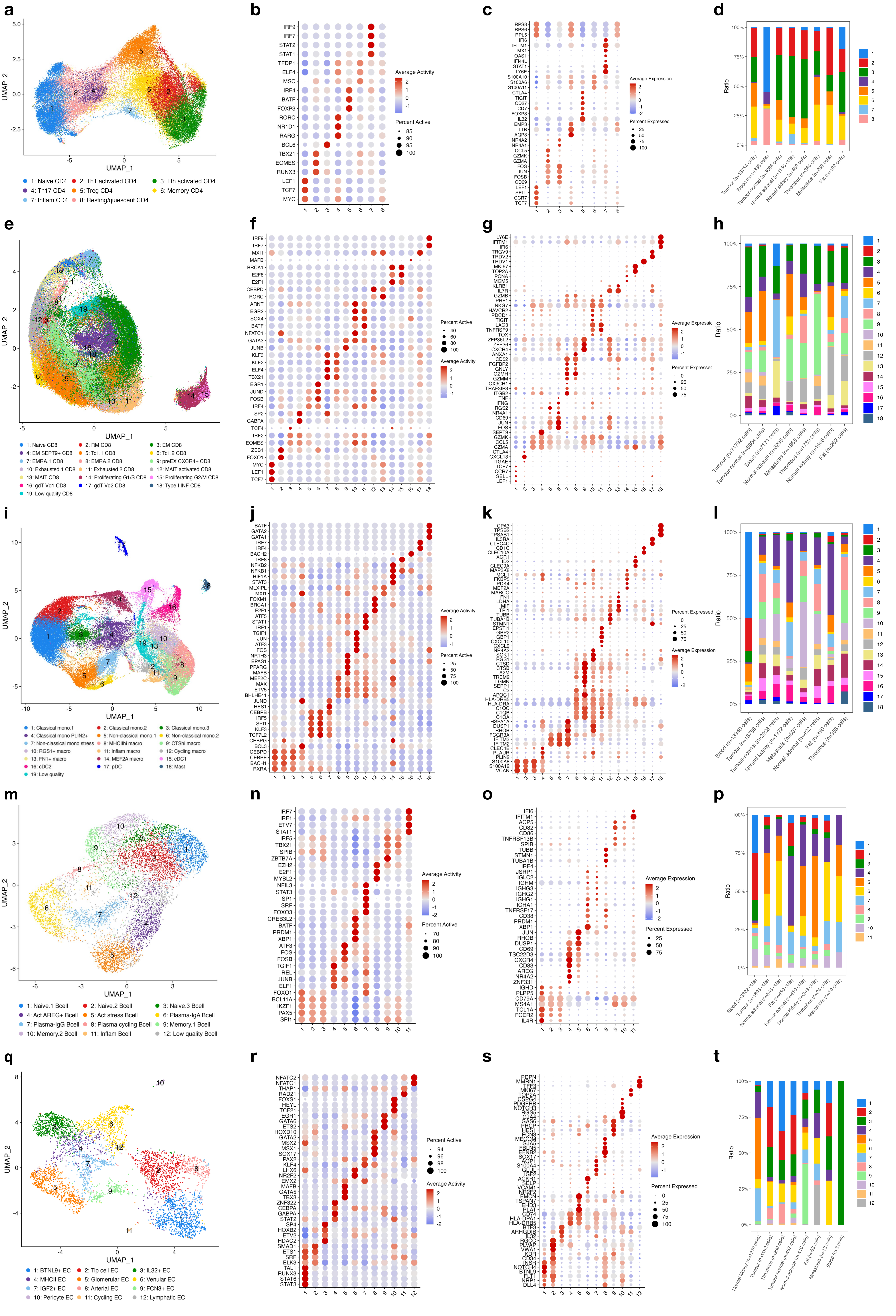
a
b
c
d
UMAP_1
UMAP_2
1: Naive CD4
2: Th1 activated CD4
3: Tfh activated CD4
4: Th17 CD4
5: Treg CD4
6: Memory CD4
7: Inflam CD4
8: Resting/quiescent CD4
Average Activity
Percent Active
Average Expression
Percent Expressed
Ratio
Tumour (n=18754 cells)
Blood (n=14338 cells)
Tumour-normal (n=3086 cells)
Normal adrenal (n=1156 cells)
Normal kidney (n=459 cells)
Thrombus (n=366 cells)
Metastasis (n=259 cells)
Fat (n=192 cells)
e
f
g
h
1: Naive CD8
2: RM CD8
3: EM CD8
4: EM SEPT9+ CD8
5: Tc1.1 CD8
6: Tc1.2 CD8
7: EMRA.1 CD8
8: EMRA.2 CD8
9: preEX CXCR4+ CD8
10: Exhausted.1 CD8
11: Exhausted.2 CD8
12: MAIT activated CD8
13: MAIT CD8
14: Proliferating G1/S CD8
15: Proliferating G2/M CD8
16: gdT Vd1 CD8
17: gdT Vd2 CD8
18: Type I INF CD8
19: Low quality CD8
Tumour (n=71792 cells)
Tumour-normal (n=8804 cells)
Blood (n=7171 cells)
Normal adrenal (n=3295 cells)
Metastasis (n=1985 cells)
Thrombus (n=1739 cells)
Normal kidney (n=1666 cells)
Fat (n=262 cells)
i
j
k
l
1: Classical mono.1
2: Classical mono.2
3: Classical mono.3
4: Classical mono PLIN2+
5: Non-classical mono.1
6: Non-classical mono.2
7: Non-classical mono stress
8: MHCIIhi macro
9: CTShi macro
10: RGS1+ macro
11: Inflam macro
12: Cycling macro
13: FN1+ macro
14: MEF2A macro
15: cDC1
16: cDC2
17: pDC
18: Mast
19: Low quality
Blood (n=18940 cells)
Tumour (n=18758 cells)
Tumour-normal (n=2928 cells)
Normal kidney (n=1372 cells)
Metastasis (n=507 cells)
Normal adrenal (n=422 cells)
Fat (n=390 cells)
Thrombus (n=358 cells)
m
n
o
p
1: Naive.1 Bcell
2: Naive.2 Bcell
3: Naive.3 Bcell
4: Act AREG+ Bcell
5: Act stress Bcell
6: Plasma-IgA Bcell
7: Plasma-IgG Bcell
8: Plasma cycling Bcell
9: Memory.1 Bcell
10: Memory.2 Bcell
11: Inflam Bcell
12: Low quality Bcell
Blood (n=3322 cells)
Tumour (n=1608 cells)
Normal adrenal (n=545 cells)
Fat (n=450 cells)
Tumour-normal (n=410 cells)
Normal kidney (n=243 cells)
Thrombus (n=26 cells)
Metastasis (n=10 cells)
q
r
s
t
1: BTNL9+ EC
2: Tip cell EC
3: IL32+ EC
4: MHCII EC
5: Glomerular EC
6: Venular EC
7: IGF2+ EC
8: Arterial EC
9: FCN3+ EC
10: Pericyte EC
11: Cycling EC
12: Lymphatic EC
Normal kidney (n=1279 cells)
Tumour (n=1192 cells)
Thrombus (n=950 cells)
Tumour-normal (n=457 cells)
Normal adrenal (n=416 cells)
Fat (n=68 cells)
Metastasis (n=13 cells)
Blood (n=3 cells)

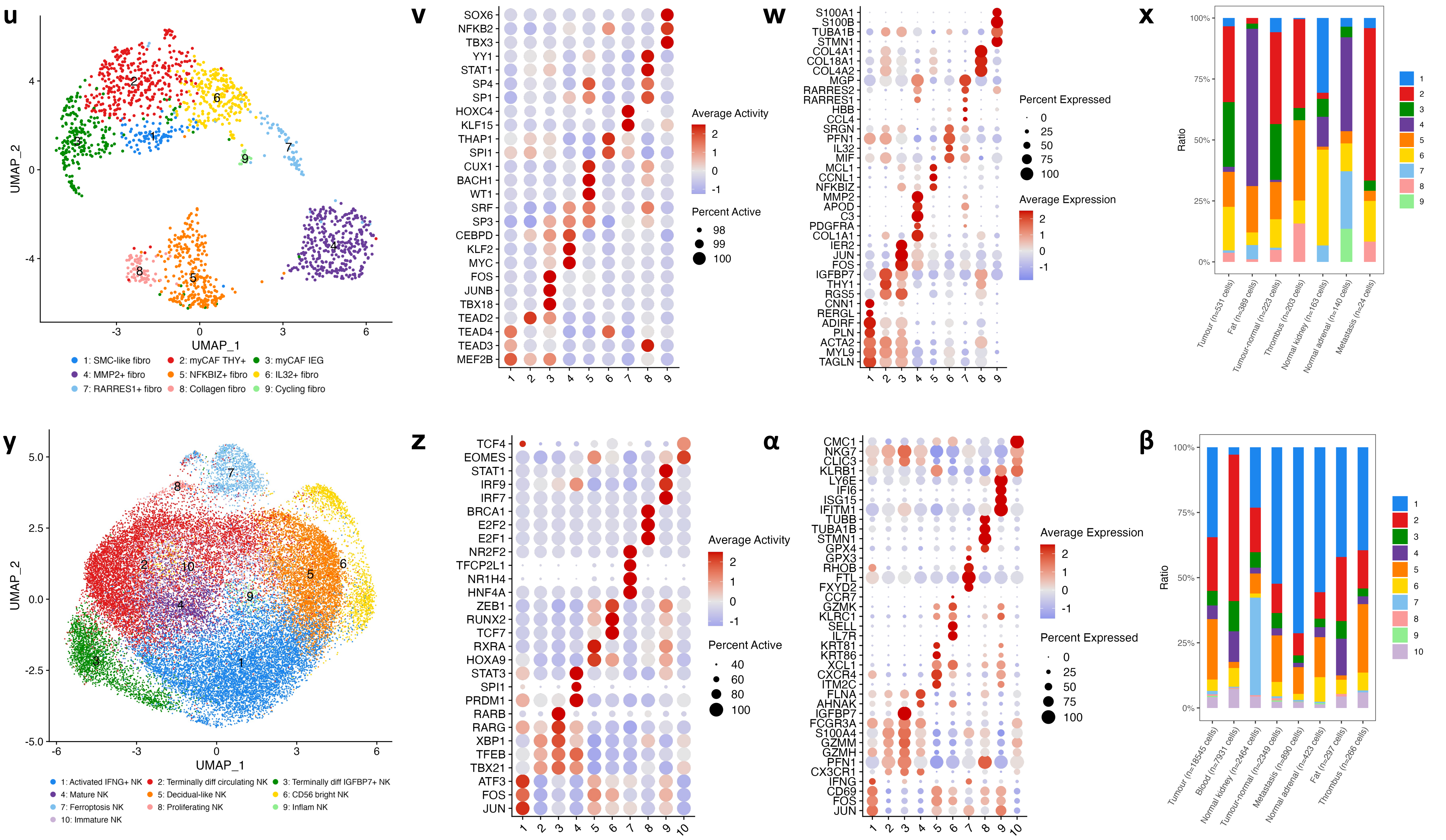

Supplementary Figure 3: Transcription factor activity-based sub-clustering of CD4, CD8, myeloid, B, endothelial, fibroblast, and NK cell compartments.

a, e, I, m, q, u, y UMAPs depicting CD4, CD8, myeloid, B, endothelial, fibroblast, and NK cell sub-clustering. **b, f, j, n, r, v, z** Regulon (TF activity) scores across the identified sub-populations. **c, g, k, o, s, w, α** Canonical marker gene expression plots. **d, h, l, p, t, x, β** Sub-cluster distributions across anatomical regions sampled for sequencing.

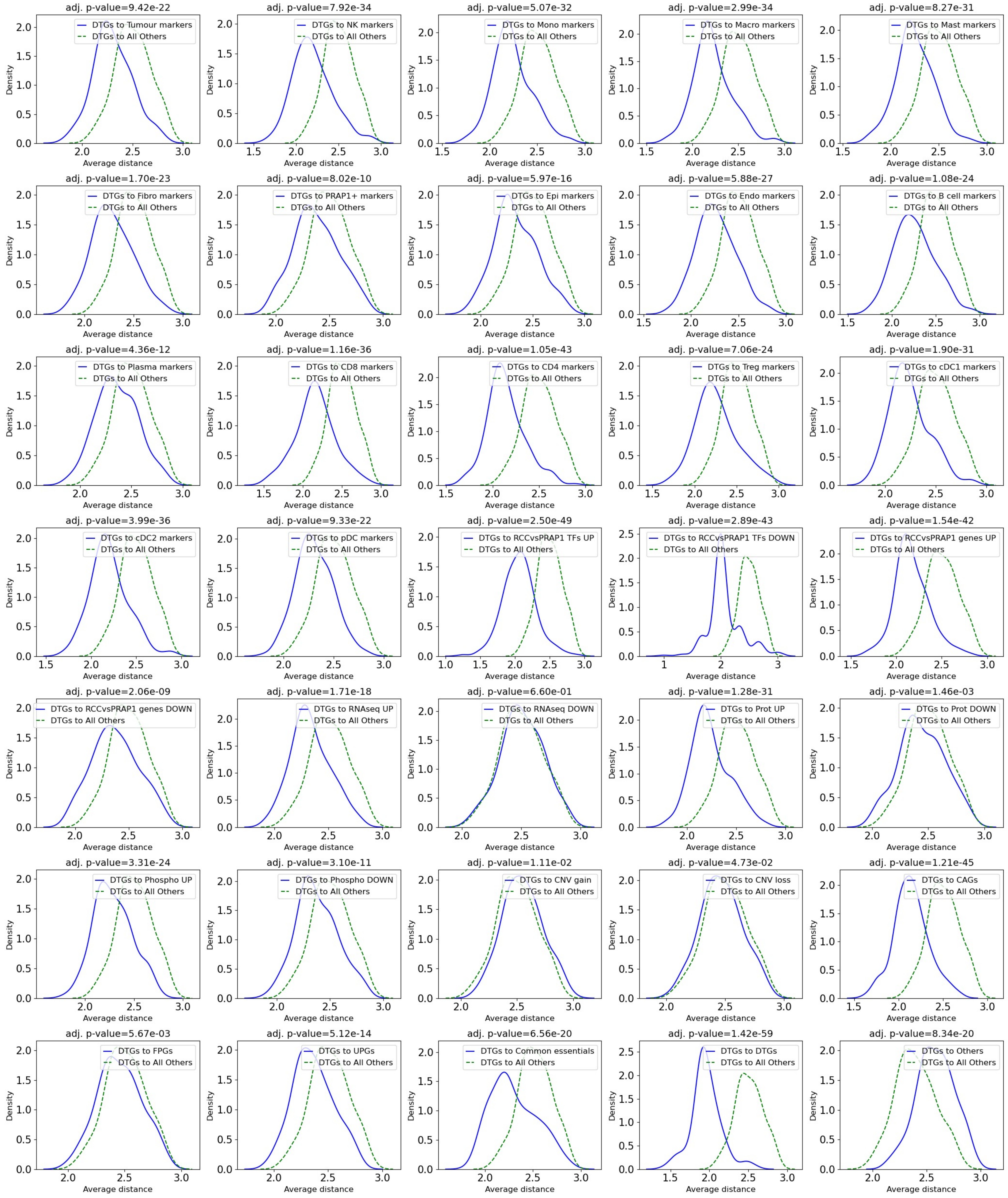

Supplementary Figure 4: Proximity of drug target genes to curated signatures within the PPI network. Average shortest path distance distributions within the PPI network are compared to assess the proximity of drug target genes (DTGs) to each curated gene signature (blue) relative to all remaining network nodes (green). Statistical significance was assessed using BH-adjusted Wilcoxon rank-sum tests; adjusted *p*-values are indicated.

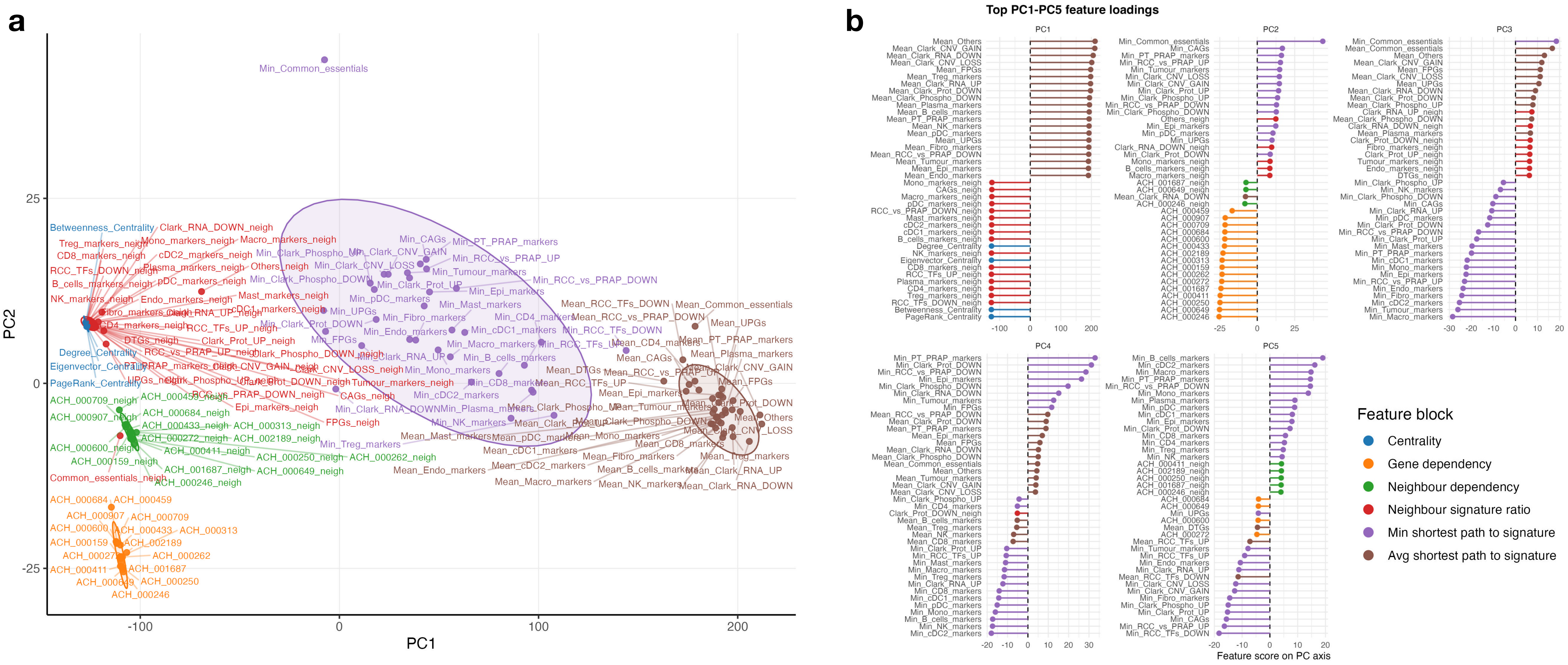

Supplementary Figure 5: Principal component analysis of the 139-dimensional gene embedding.
a Projection of features onto the first two principal components (PC1-PC2) of the gene embedding, with features coloured by their predefined blocks. **b** Top positive and negative feature loadings for principal components 1-5.

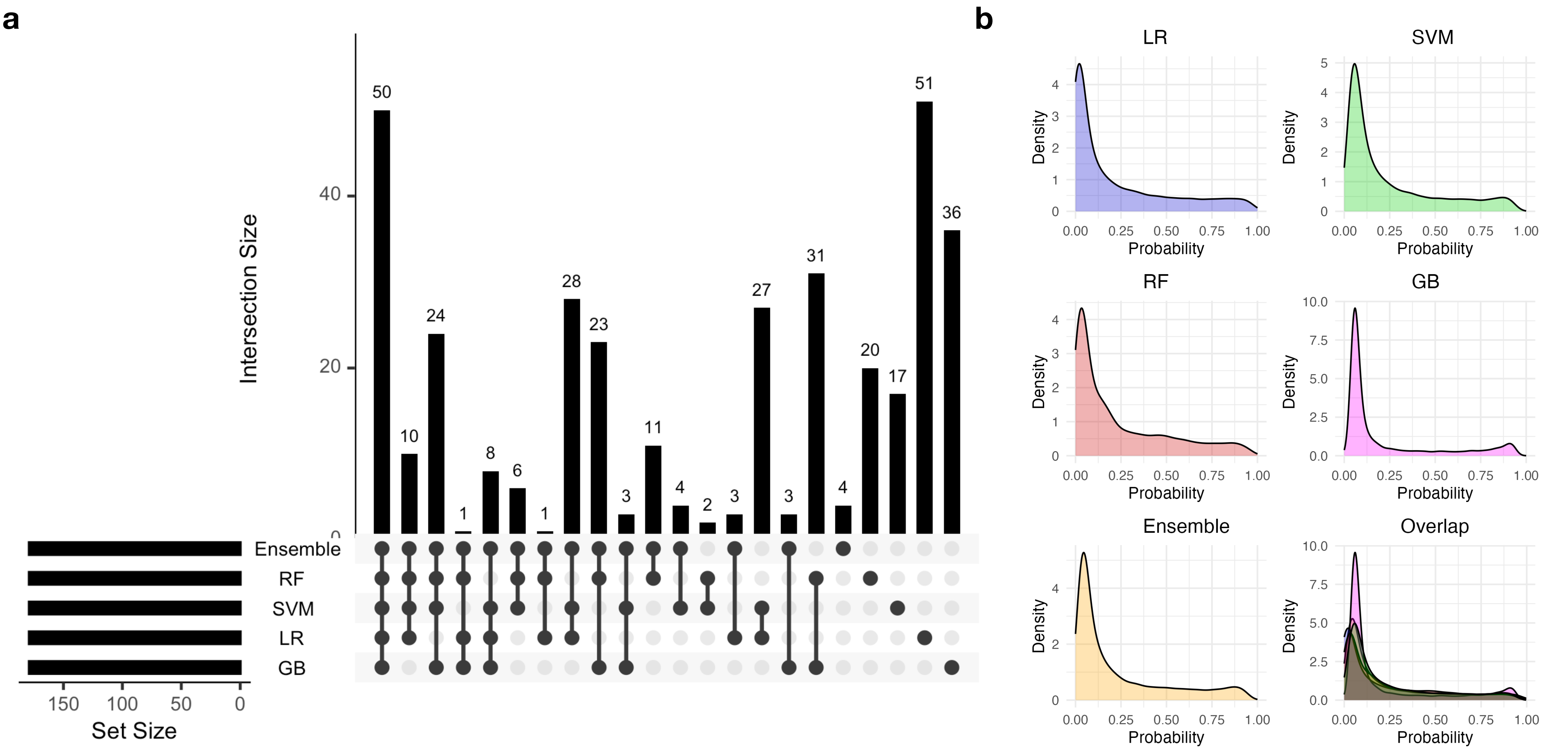

Supplementary Figure 6: Prediction consistency across machine learning classifiers.
a Overlap of the top 2% of predictions across ML classifiers. **b** Class 1 probability density distributions across the applied ML classifiers.

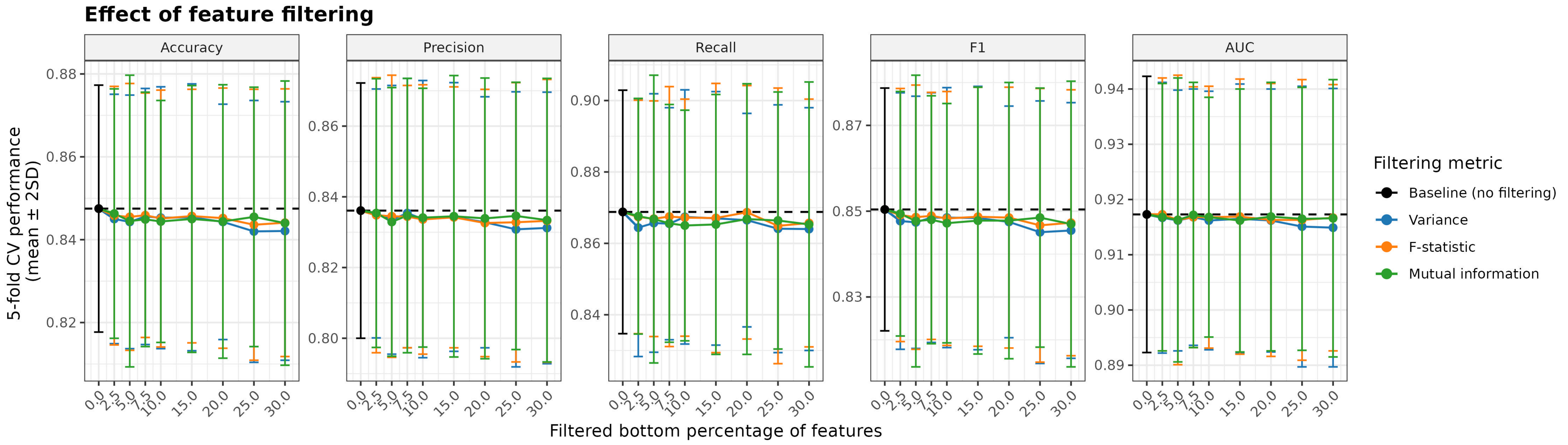

Supplementary Figure 7: Effect of feature filtering on model performance.
Five-fold cross-validation performance (mean ± 2 SD) as a function of progressively removing the lowest-ranked 2.5-30% of features based on variance, F-statistic, or mutual information with the class label. Panels correspond to different ML performance metrics.

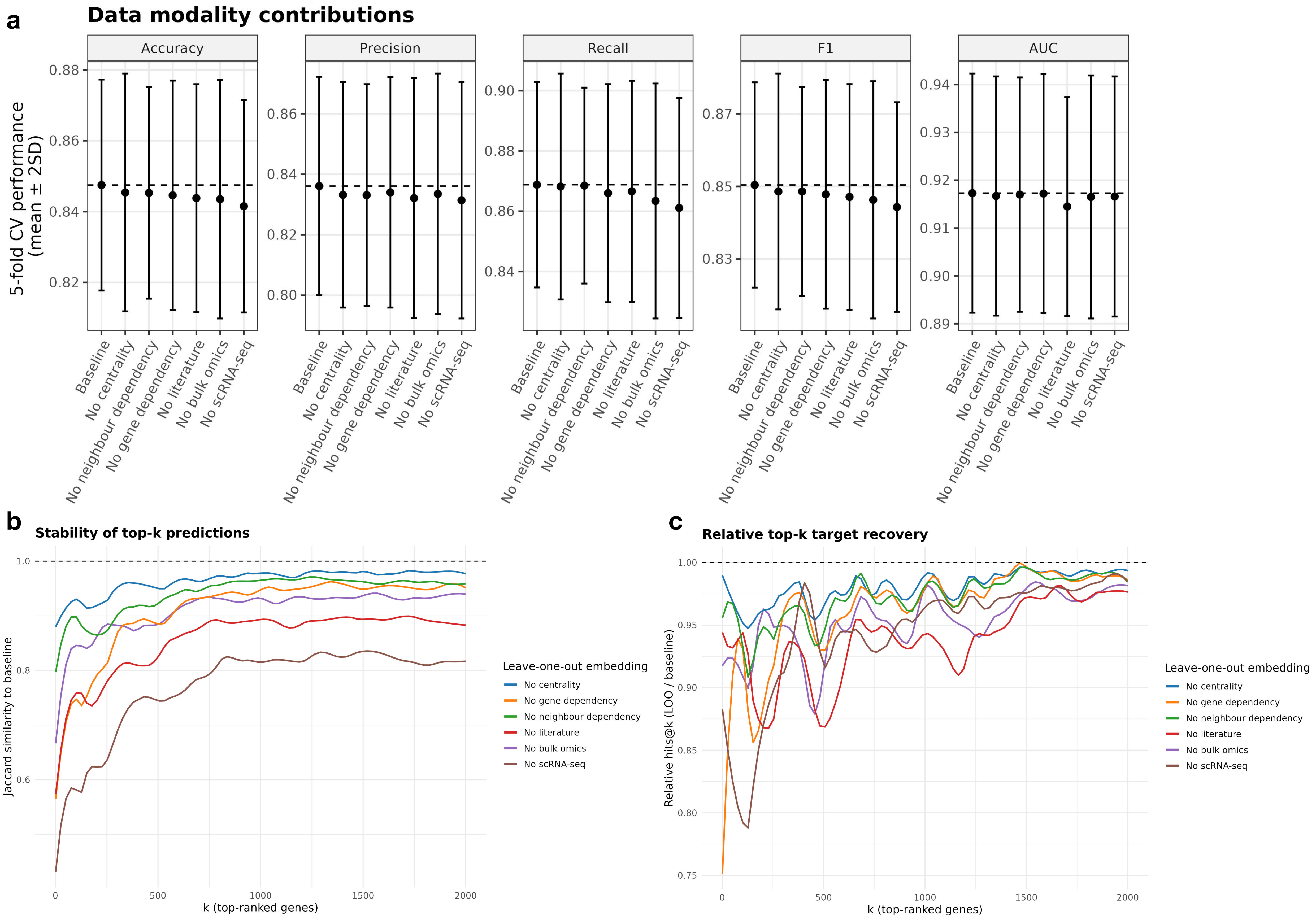

Supplementary Figure 8: Data modality ablation analysis.
a Five-fold cross-validation performance (mean ± 2 SD) of the baseline model and leave-one-modality-out embeddings, showing the effect of removing network centrality features; CRISPR gene-level or neighbour-level dependency features; literature-derived gene sets (DTGs, common essential genes, as well as gene lists derived from DisGeNET and Human Protein Atlas databases); bulk-omics features; or scRNA-seq features across ML performance metrics. **b** Loess-smoothed Jaccard similarity to the baseline model as a function of the number of top-ranked genes (k), illustrating how removal of individual data modalities affects the stability of gene ranking. **c** Loess-smoothed target recovery (hits@k) relative to the baseline model as a function of the number of top-ranked genes (k), showing the impact of modality removal on recovery of known drug targets across the ranked list.

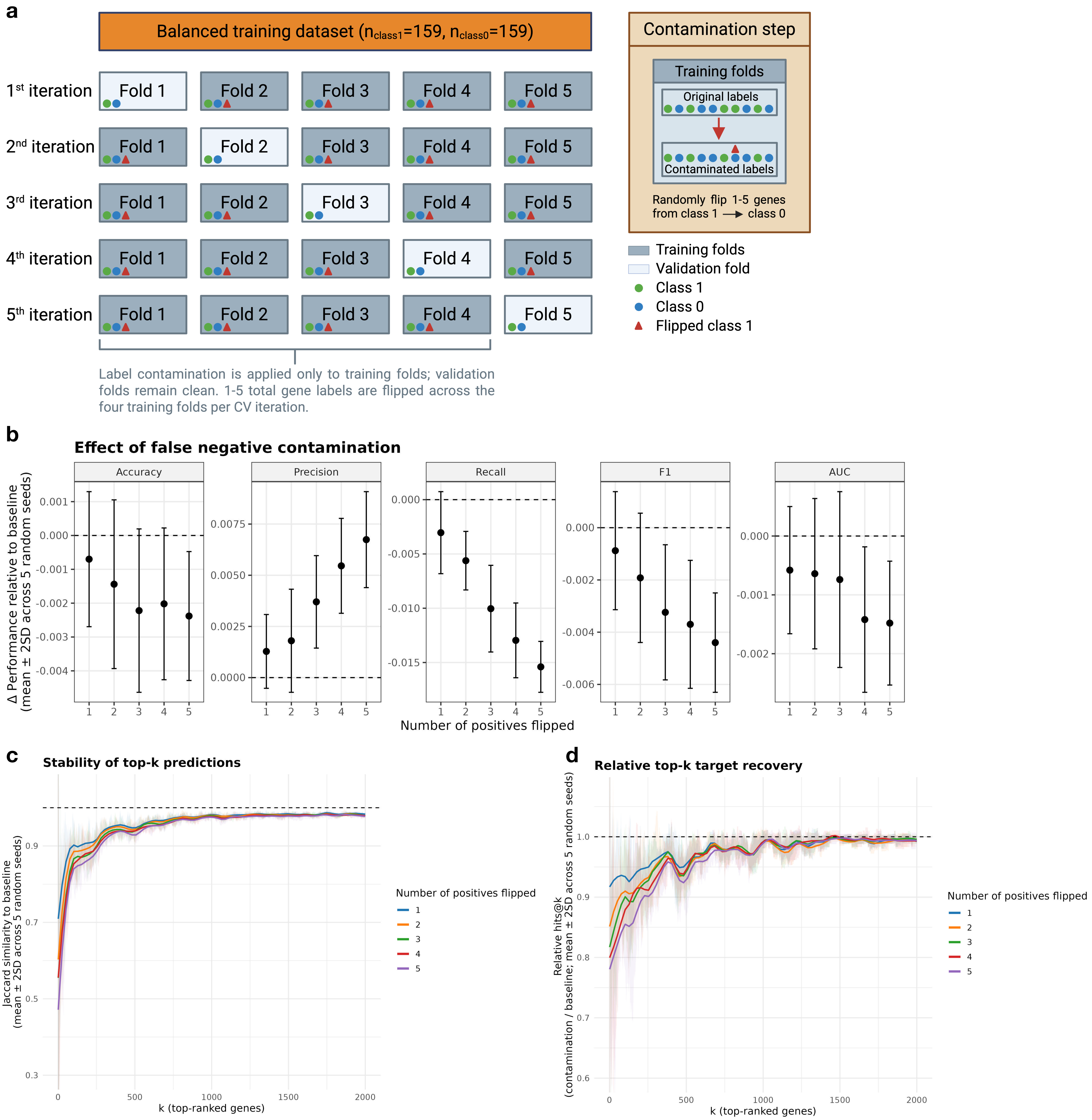

Supplementary Figure 9: Label contamination analysis.

a Schematic of the controlled false-negative label contamination strategy applied during five-fold cross-validation. The procedure is performed independently for each of the 89 balanced training datasets (Class 1 = ccRCC drug targets; Class 0 = non-targets), with each balanced dataset assigned a random seed controlling label flipping. Within each cross-validation iteration, label contamination is applied only to the four training folds, where the specified number of Class 1 gene labels are randomly flipped to Class 0 and distributed across the training folds; validation folds remain clean. To quantify variability due to stochastic label noise, the entire procedure is repeated to generate five independent contamination models per contamination level, each using a distinct random seed assignment across the 89 balanced datasets. **b** Changes in five-fold cross-validation performance relative to the baseline model (mean ± 2 SD), shown for increasing levels of false-negative label contamination. Values represent differences from the baseline embedding, with mean and variability computed across five random seeds per contamination level. **c** Mean loess-smoothed Jaccard similarity to the baseline model as a function of the number of top-ranked genes (k), shown as mean ± 2 SD across five random seeds for each contamination level, illustrating how increasing label contamination affects ranking stability. **d** Mean loess-smoothed target recovery (hits@k) relative to the baseline model as a function of the number of top-ranked genes (k), shown as mean ± 2 SD across five random seeds for each contamination level, highlighting the impact of false-negative contamination on recovery of known drug targets across the ranked list.

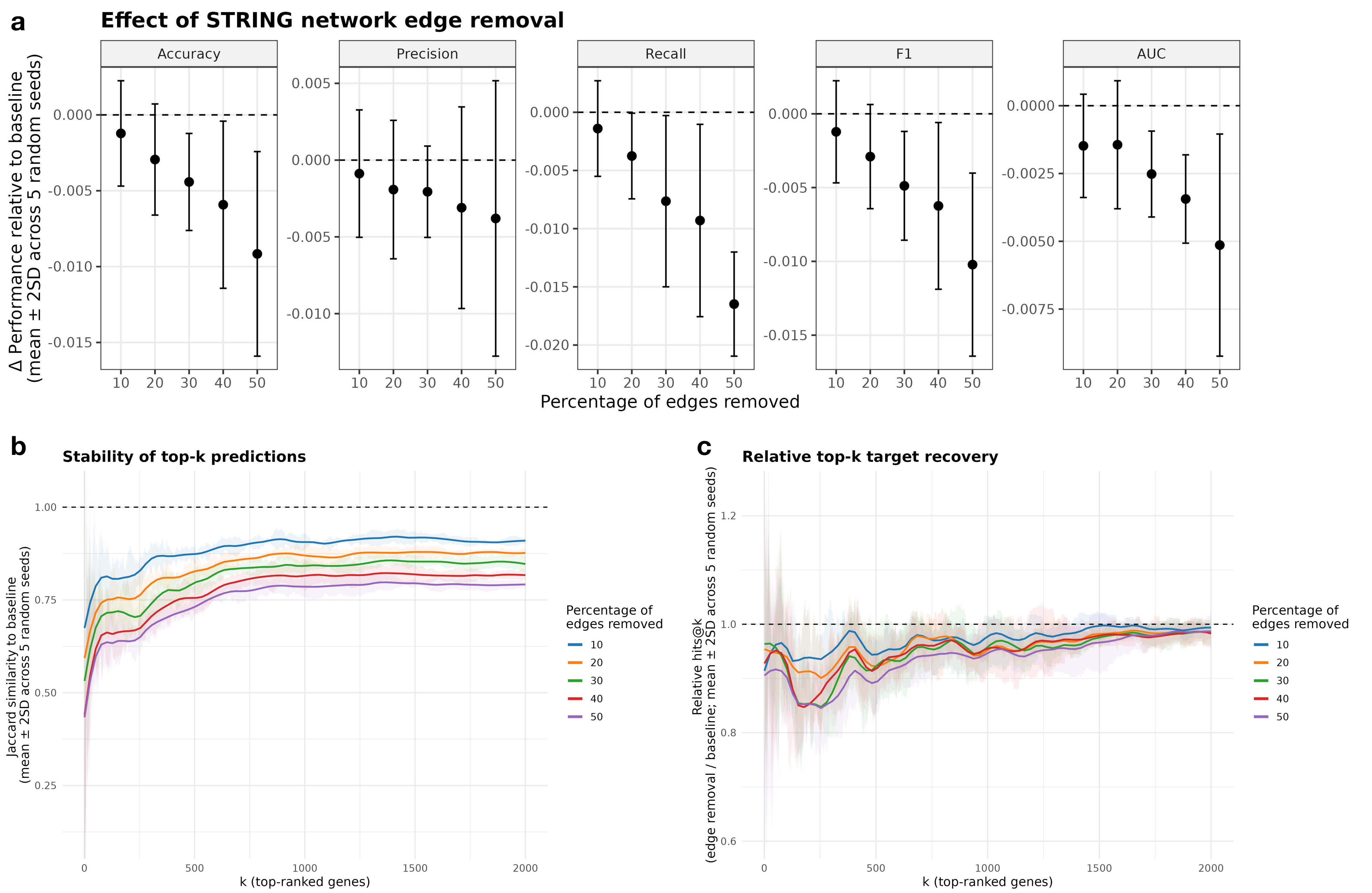

Supplementary Figure 10: STRING network edge removal analysis.
a Changes in five-fold cross-validation performance relative to the baseline model (mean ± 2 SD), shown for increasing levels of random PPI edge removal. Values represent differences from the baseline embedding, with mean and variability computed across five randomly generated networks per removal level. **b** Mean loess-smoothed Jaccard similarity to the baseline model as a function of the number of top-ranked genes (k), shown as mean ± 2 SD across five randomly generated networks for each edge removal level, illustrating how progressive network sparsification affects ranking stability. **c** Mean loess-smoothed target recovery (hits@k) relative to the baseline model as a function of the number of top-ranked genes (k), shown as mean ± 2 SD across five randomly generated networks for each edge removal level, highlighting the impact of network incompleteness on recovery of known drug targets across the ranked list.

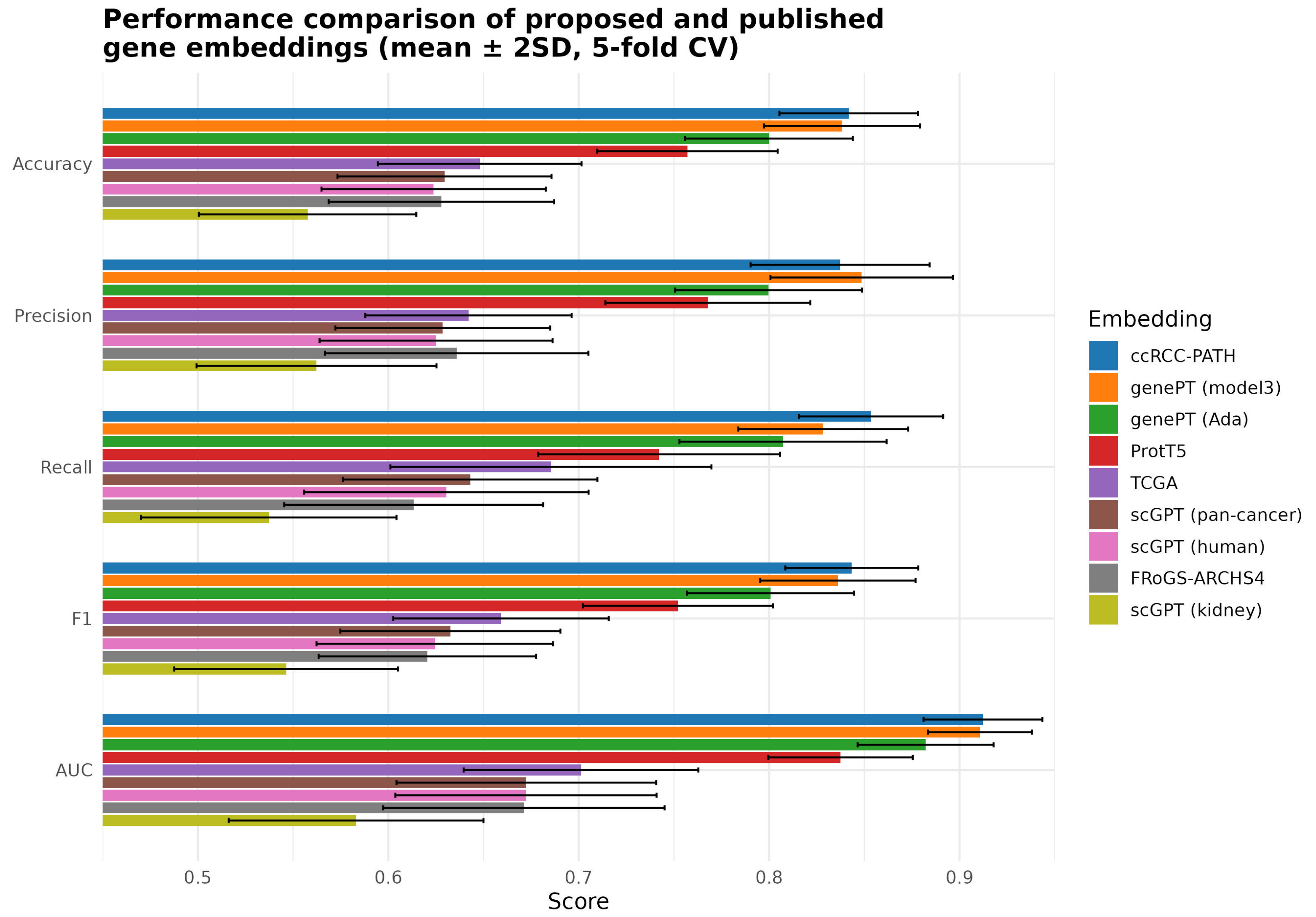

Supplementary Figure 11: Training set performance across gene embeddings.
Five-fold cross-validation performance (mean ± 2 SD) of the proposed ccRCC gene embedding and eight pre-trained gene embeddings on the training split.

a

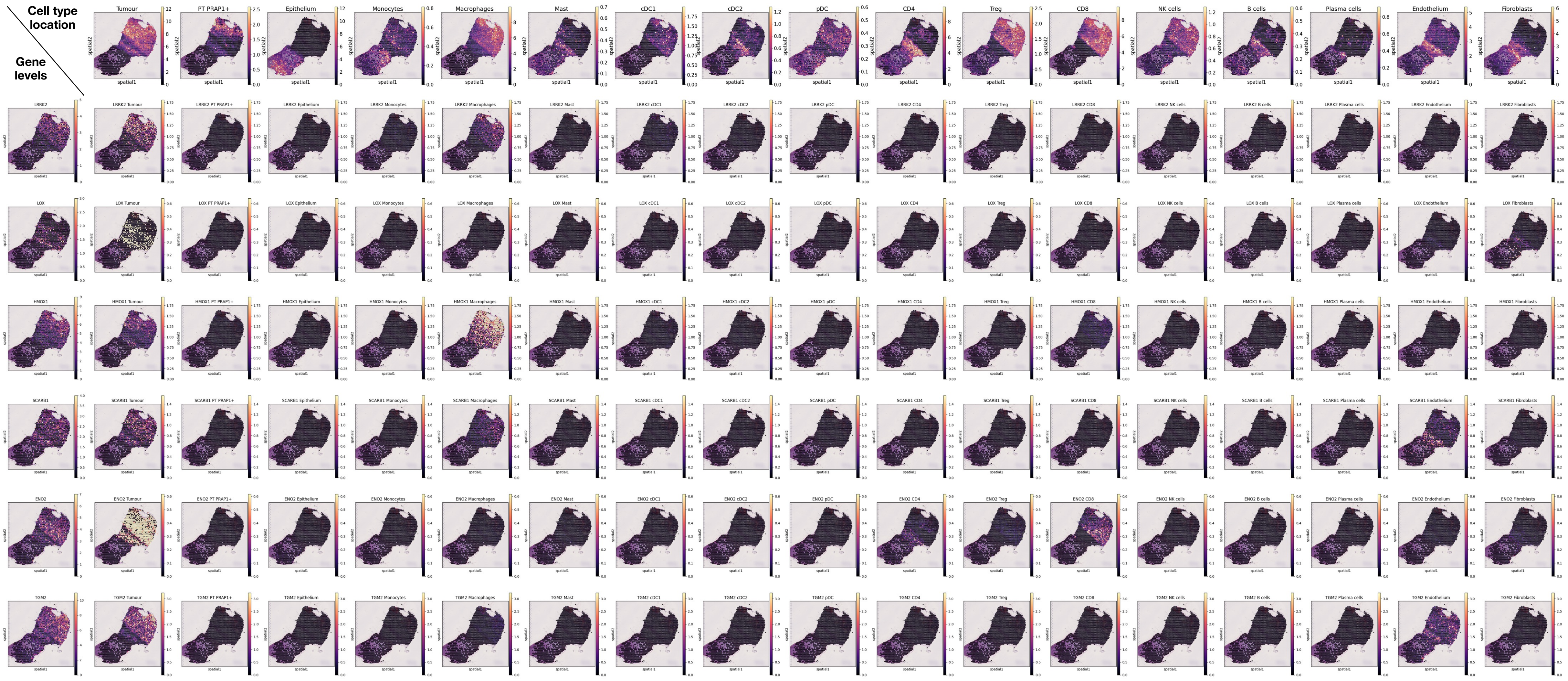

b

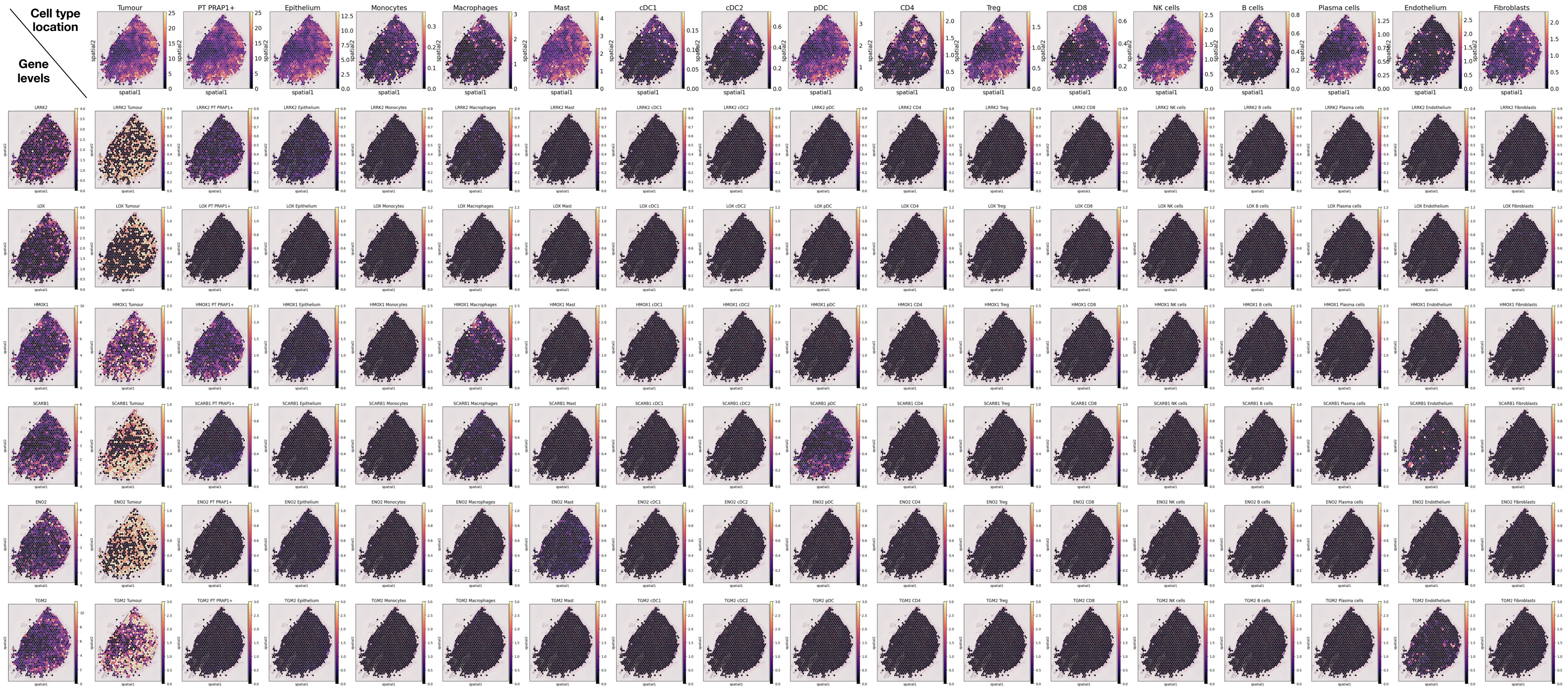

Supplementary Figure 12: Target expression patterns as observed in ccRCC tumour spatial transcriptomics data.

a ccRCC tumour-normal interface spatial transcriptomics data from patient PD47171. The top row depicts localisation of a given cell type, while the subsequent rows show gene levels across cell types. **b** ccRCC tumour core spatial transcriptomics data from patient PD43948. The top row depicts localisation of a given cell type, while the subsequent rows show gene levels across cell types.

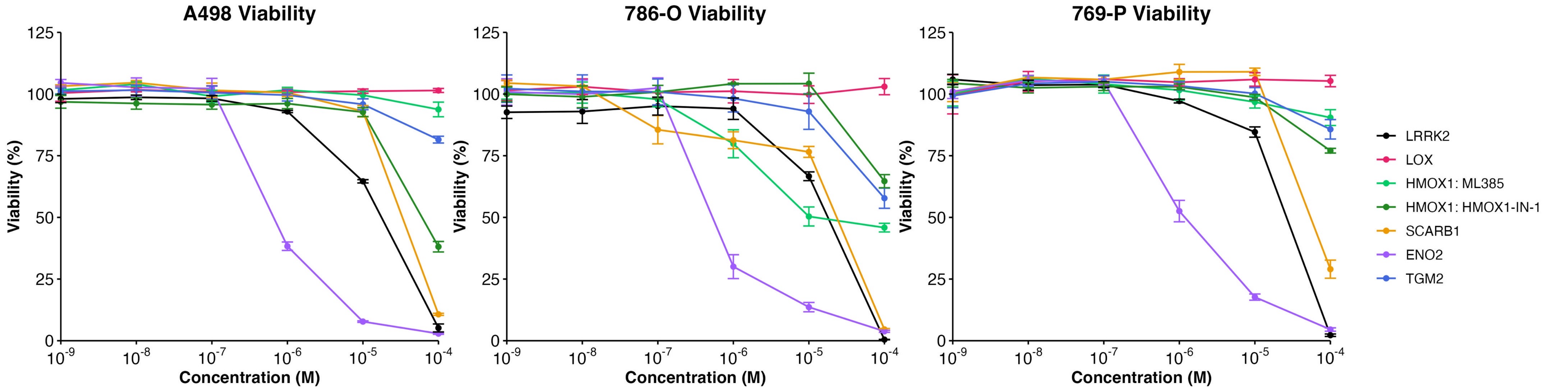

Supplementary Figure 13: Inhibitor cytotoxicity across ccRCC cell lines. A498, 786-O, and 769-P cells were treated in triplicate for 48 hours with either vehicle controls or small molecule inhibitors across six concentrations. Results are presented as mean±SD of the three technical replicates.

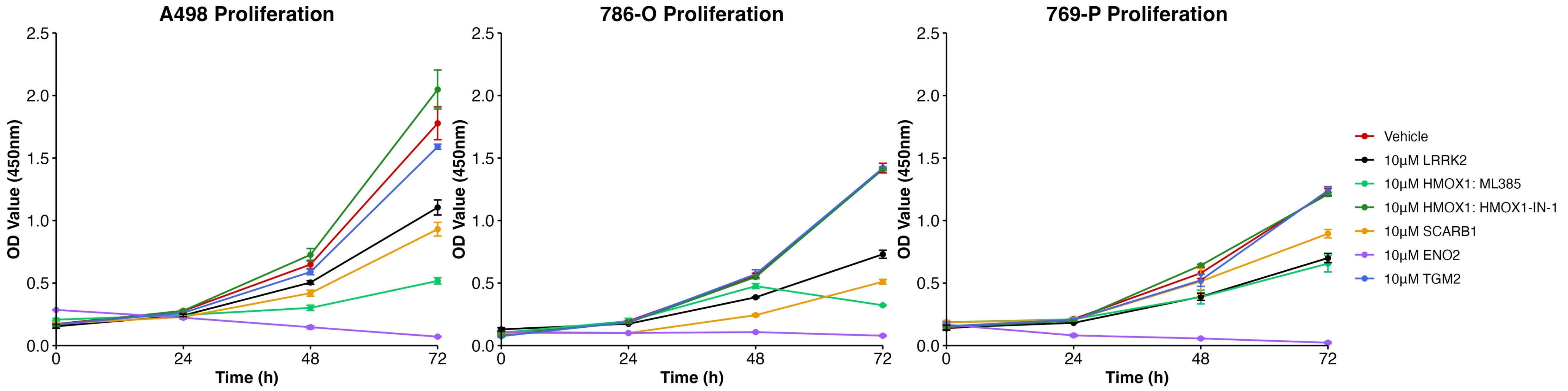

Supplementary Figure 14: Inhibitor cytostatic effects across ccRCC cell lines. A498, 786-O, and 769-P cells were treated in triplicate for 72 hours with either vehicle controls or 10µM of inhibitors targeting the genes of interest. Results are presented as mean±SD of the three technical replicates.

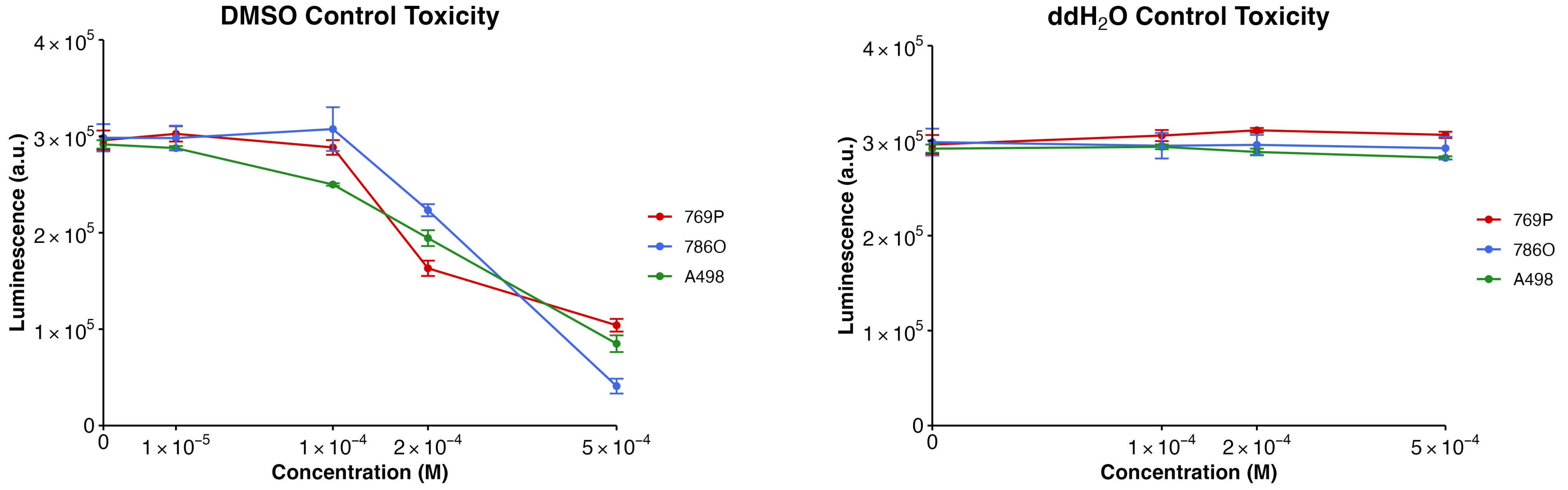

Supplementary Figure 15: DMSO and ddH2O carrier cytotoxicity across ccRCC cell lines.

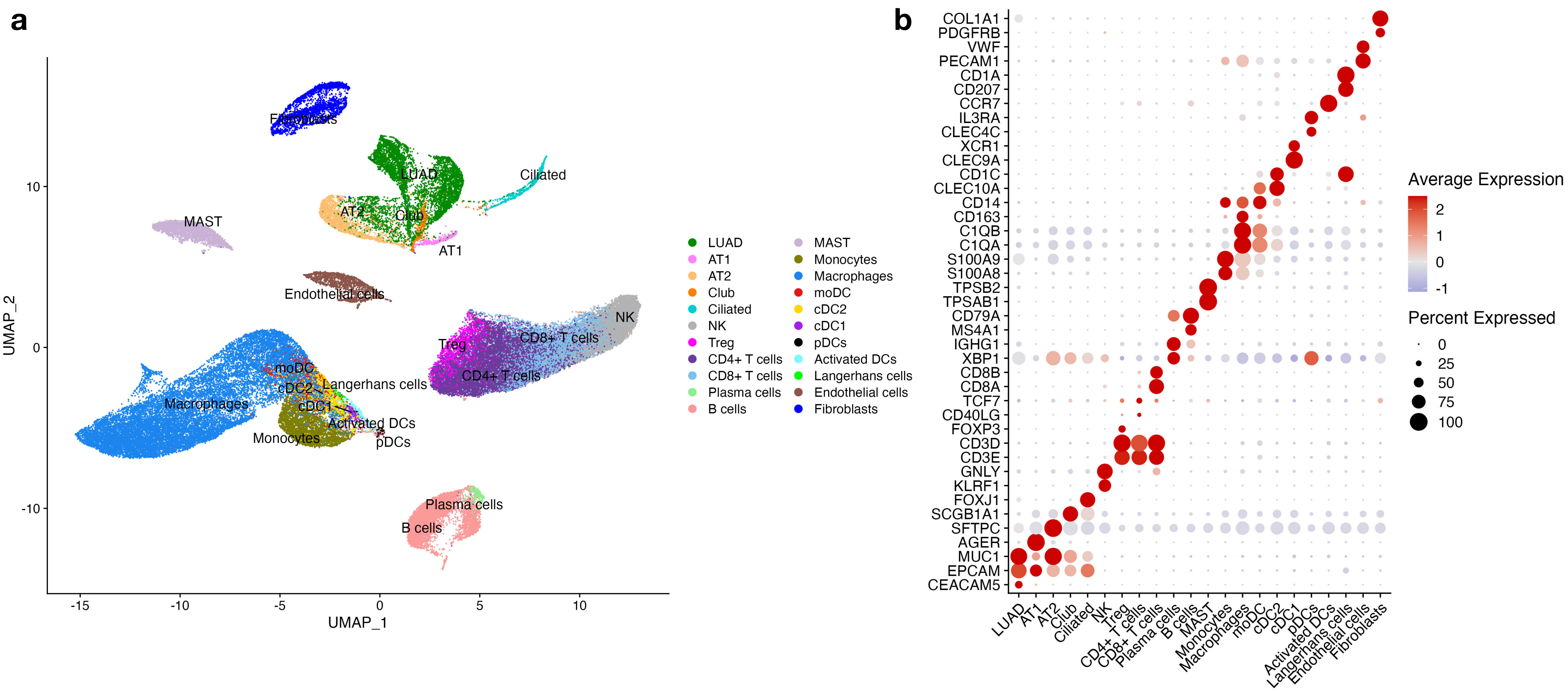

Supplementary Figure 16: Single-cell transcriptomic profiling of LUAD reveals 22 broad cell compartments.

a UMAP visualisation of 77,768 cells from 16 donors following filtering of the Kim *et al.* cohort. Cells were restricted to lung tissue as the primary site of origin and further filtered to retain only high-confidence cell-type annotations provided by the original authors. Cells are coloured by these annotations. **b** Expression of canonical marker genes used to define the major cell compartments.

Signature type	Signature data source	Description	Number of signatures
DTGs	DrugBank	Genes targeted by FDA-approved therapeutics for LUAD, or by drugs that are currently in clinical trials for LUAD (**Supplementary Data 6**).	1
CDAGs	Bulk LUAD data from Gillette et al.	Up- and down-regulated transcripts, proteins, phosphoproteins and acetylproteins in tumour vs healthy tissue comparisons, as identified through bulk RNA-seq, proteomics, phosphoproteomics and acetylproteomics differential abundance analysis; Gene copy number gains and losses identified through WGS data analysis.	10
	scRNA-seq tumour vs epithelium DEGs	Up- and down-regulated genes in scRNA-seq tumour vs epithelial AT1/AT2/ciliated/club cell differential gene expression analysis.	8
	scRNA-seq broad cell type markers	Up-regulated genes in scRNA-seq differential expression analysis comparing tumour cells, AT1, AT2, club, ciliated, NK, CD4+ T cells, Tregs, CD8+ T cells, B cells, plasma cells, mast cells, monocytes, macrophages, moDCs, cDC1, cDC2, pDCs, activated DCs, Langerhans cells, endothelium, and fibroblasts.	22
	DisGeNET	LUAD-associated genes (LAGs).	1
	Human Protein Atlas	Favourable and unfavourable LUAD prognosis genes (FPGs and UPGs).	2
	DepMap	Genes classified as common essentials by DepMap CRISPR or RNAi projects.	1
	'Others'	Genes that do not belong to any of the gene sets outlined above.	1
		Total	46

Supplementary Table 1: LUAD drug target and candidate disease-associated gene signature descriptions.

Metric	Ensemble	GB	LR	RF	SVM
Accuracy	0.8252±0.0288	0.8232±0.0310	0.8051±0.0390	0.8212±0.0262	0.8200±0.0330
Precision	0.8294±0.0356	0.8285±0.0385	0.8027±0.0451	0.8311±0.0378	0.8256±0.0430
Recall	0.8233±0.0343	0.8195±0.0405	0.8145±0.0399	0.8108±0.0357	0.8166±0.0475
F1	0.8245±0.0291	0.8220±0.0318	0.8067±0.0378	0.8188±0.0265	0.8189±0.0335
AUC	0.9047±0.0248	0.9019±0.0276	0.8897±0.0330	0.9004±0.0245	0.8935±0.0286

Supplementary Table 2: LUAD performance metrics across 5 ML classifiers obtained using 5-fold CV on the training data.

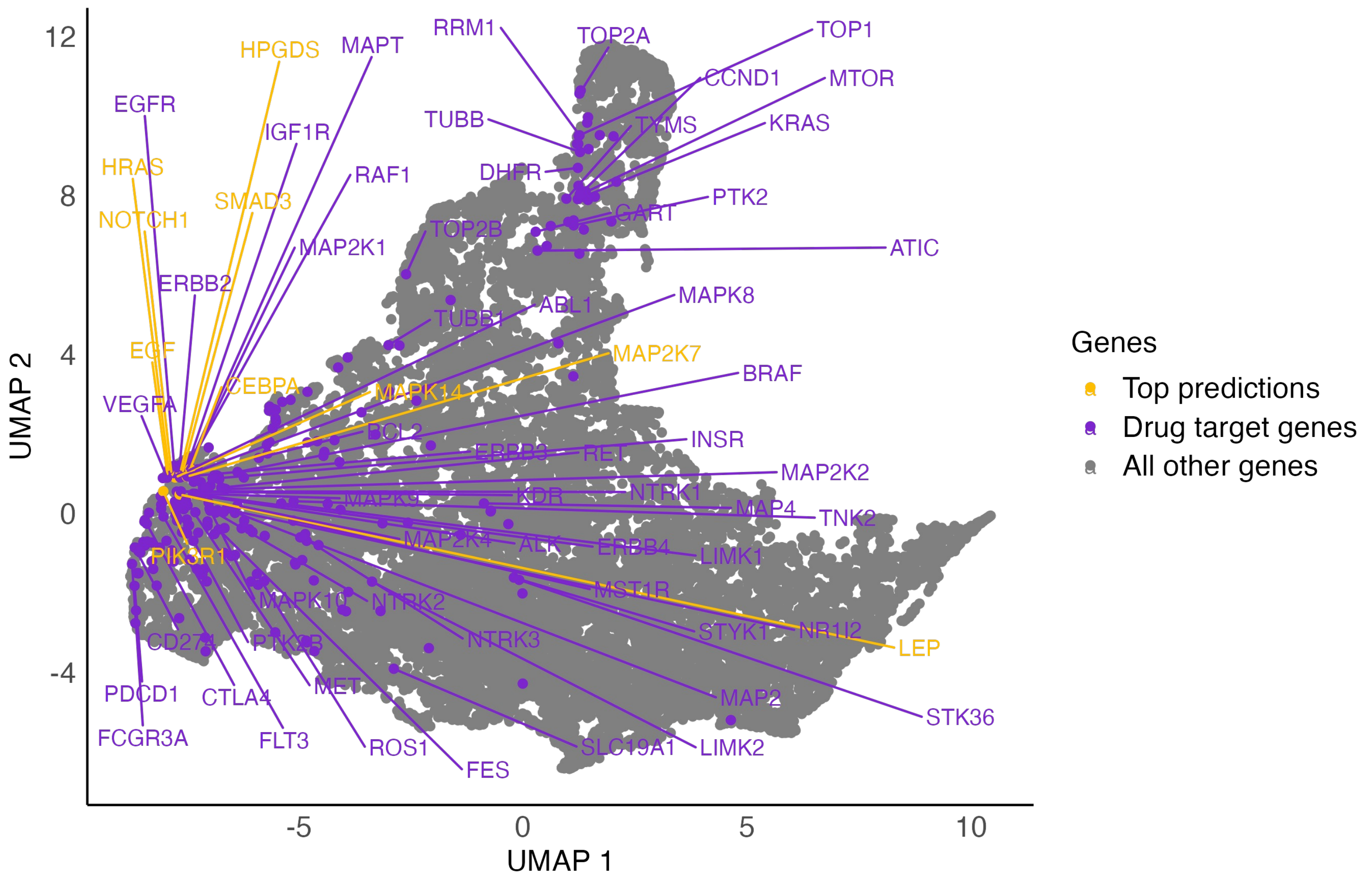

Supplementary Figure 17: UMAP visualisation of the LUAD feature space.
UMAP projection of the LUAD gene embedding used for model training and evaluation (17,880 genes; 246 features). Genes in the LUAD drug target gene (DTG) set are highlighted in purple; FDA-approved LUAD targets are labelled, while targets currently under clinical investigation are shown without labels. The unrefined set of top model predictions is highlighted and labelled in yellow.
