# Supplementary material for: A network medicine framework for multi-modal data integration in therapeutic target discovery
Source: Commun Chem. 2026 May 6;9:244. doi: 10.1038/s42004-026-02049-9 (PMC13358115; doi:10.1038/s42004-026-02049-9)
Supplement: Supplementary file 11 — nr-reporting-summary_v1 [file 42004_2026_2049_MOESM11_ESM.pdf]

Corresponding author(s): Kourosh Saeb-Parsy, Namshik Han

Last updated by author(s): Mar 27, 2026

Reporting Summary

Nature Portfolio wishes to improve the reproducibility of the work that we publish. This form provides structure for consistency and transparency in reporting. For further information on Nature Portfolio policies, see our Editorial Policies and the Editorial Policy Checklist.

Statistics

For all statistical analyses, confirm that the following items are present in the figure legend, table legend, main text, or Methods section.

n/a	Confirmed	
☐	☒	The exact sample size (*n*) for each experimental group/condition, given as a discrete number and unit of measurement
☐	☒	A statement on whether measurements were taken from distinct samples or whether the same sample was measured repeatedly
☐	☒	The statistical test(s) used AND whether they are one- or two-sided<br>*Only common tests should be described solely by name; describe more complex techniques in the Methods section.*
☒	☐	A description of all covariates tested
☐	☒	A description of any assumptions or corrections, such as tests of normality and adjustment for multiple comparisons
☐	☒	A full description of the statistical parameters including central tendency (e.g. means) or other basic estimates (e.g. regression coefficient) AND variation (e.g. standard deviation) or associated estimates of uncertainty (e.g. confidence intervals)
☐	☒	For null hypothesis testing, the test statistic (e.g. *F*, *t*, *r*) with confidence intervals, effect sizes, degrees of freedom and *P* value noted<br>*Give P values as exact values whenever suitable.*
☒	☐	For Bayesian analysis, information on the choice of priors and Markov chain Monte Carlo settings
☒	☐	For hierarchical and complex designs, identification of the appropriate level for tests and full reporting of outcomes
☐	☒	Estimates of effect sizes (e.g. Cohen's *d*, Pearson's *r*), indicating how they were calculated

Our web collection on statistics for biologists contains articles on many of the points above.

Software and code

Policy information about availability of computer code

Data collection	All statistical analyses were performed using R (v4.3.1, http://www.r-project.org/). The code for gene embedding construction and machine learning analyses is available at GitHub (https://github.com/Greta-B/NetworkTargetID) and Zenodo (https://doi.org/10.5281/zenodo.19240170) repositories.
Data analysis	All statistical analyses were performed using R (v4.3.1, http://www.r-project.org/). The code for gene embedding construction and machine learning analyses is available at GitHub (https://github.com/Greta-B/NetworkTargetID) and Zenodo (https://doi.org/10.5281/zenodo.19240170) repositories.

For manuscripts utilizing custom algorithms or software that are central to the research but not yet described in published literature, software must be made available to editors and reviewers. We strongly encourage code deposition in a community repository (e.g. GitHub). See the Nature Portfolio guidelines for submitting code & software for further information.

Data

Policy information about availability of data

All manuscripts must include a data availability statement. This statement should provide the following information, where applicable:

- Accession codes, unique identifiers, or web links for publicly available datasets
- A description of any restrictions on data availability
- For clinical datasets or third party data, please ensure that the statement adheres to our policy

The ccRCC scRNA-seq and spatial transcriptomics datasets are available from Mendeley Data (https://data.mendeley.com/datasets/g67bkbnhhg/1). Bulk ccRCC omics data are available from the CPTAC data portal (https://cptac-data-portal.georgetown.edu/cptac/s/S050). CRISPR gene dependency data are available from the DepMap portal (https://depmap.org; 23Q4 release). LUAD scRNA-seq dataset is available from GEO (GSE131907).

Research involving human participants, their data, or biological material

Policy information about studies with human participants or human data. See also policy information about sex, gender (identity/presentation), and sexual orientation and race, ethnicity and racism.

Reporting on sex and gender	This study did not involve human participants, human data, or human biological materials.
Reporting on race, ethnicity, or other socially relevant groupings	This study did not involve human participants, human data, or human biological materials.
Population characteristics	This study did not involve human participants, human data, or human biological materials.
Recruitment	This study did not involve human participants, human data, or human biological materials.
Ethics oversight	This study did not involve human participants, human data, or human biological materials.

Note that full information on the approval of the study protocol must also be provided in the manuscript.

Field-specific reporting

Please select the one below that is the best fit for your research. If you are not sure, read the appropriate sections before making your selection.

☒ Life sciences ☐ Behavioural & social sciences ☐ Ecological, evolutionary & environmental sciences

For a reference copy of the document with all sections, see nature.com/documents/nr-reporting-summary-flat.pdf

Life sciences study design

All studies must disclose on these points even when the disclosure is negative.

Sample size	Sample size was determined based on previous publications and consistency across biological replicates.
Data exclusions	No data were excluded unless technical failure was confirmed.
Replication	Each experiment was replicated three times independently with consistent results.
Randomization	No randomization was used.
Blinding	Blinding was also not performed.

Reporting for specific materials, systems and methods

We require information from authors about some types of materials, experimental systems and methods used in many studies. Here, indicate whether each material, system or method listed is relevant to your study. If you are not sure if a list item applies to your research, read the appropriate section before selecting a response.

Materials & experimental systems

n/a	Involved in the study
☒	☐ Antibodies
☐	☒ Eukaryotic cell lines
☒	☐ Palaeontology and archaeology
☒	☐ Animals and other organisms
☒	☐ Clinical data
☒	☐ Dual use research of concern
☒	☐ Plants

Methods

n/a	Involved in the study
☒	☐ ChIP-seq
☒	☐ Flow cytometry
☒	☐ MRI-based neuroimaging

Eukaryotic cell lines

Policy information about cell lines and Sex and Gender in Research

Cell line source(s)	ccRCC cell lines were kindly provided by the Sakari Vanharanta laboratory.
Authentication	The identity of the cell lines was confirmed by short-tandem repeat analysis (see Vanharanta 2026 (PMID: 41420106), Vanharanta 2023 (PMID: 37554444)).
Mycoplasma contamination	Cell lines tested negative for mycoplasma contamination (see Vanharanta 2026 (PMID: 41420106), Vanharanta 2023 (PMID: 37554444)).
Commonly misidentified lines (See ICLAC register)	We did not use any misidentified cell lines.

Plants

Seed stocks	N/A
Novel plant genotypes	N/A
Authentication	N/A
